# Coherent spin-control of *S* = 1 vanadium and molybdenum complexes†

**DOI:** 10.1039/d4sc03107e

**Published:** 2024-08-05

**Authors:** Daniel W. Laorenza, Kathleen R. Mullin, Leah R. Weiss, Sam L. Bayliss, Pratiti Deb, David D. Awschalom, James M. Rondinelli, Danna E. Freedman

**Affiliations:** a Department of Chemistry, Massachusetts Institute of Technology Cambridge Massachusetts 02139 USA danna@mit.edu; b Department of Materials Science and Engineering, Northwestern University Evanston Illinois 60208 USA jrondinelli@northwestern.edu; c Pritzker School of Molecular Engineering, University of Chicago Chicago Illinois 60637 USA awsch@uchicago.edu; d Advanced Institute for Materials Research (AIMR-WPI), Tohoku University Sendai 980-8577 Japan; e James Watt School of Engineering, University of Glasgow Glasgow G12 8QQ UK; f Department of Physics, University of Chicago Chicago Illinois 60637 USA; g Center for Molecular Engineering and Materials Science Division, Argonne National Laboratory Lemont Illinois 60439 USA

## Abstract

The burgeoning field of quantum sensing hinges on the creation and control of quantum bits. To date, the most well-studied quantum sensors are optically active, paramagnetic defects residing in crystalline hosts. We previously developed analogous optically addressable molecules featuring a ground-state spin-triplet centered on a Cr^4+^ ion with an optical-spin interface. In this work, we evaluate isovalent V^3+^ and Mo^4+^ congeners, which offer unique advantages, such as an intrinsic nuclear spin for V^3+^ or larger spin–orbit coupling for Mo^4+^, as optically addressable spin systems. We assess the ground-state spin structure and dynamics for each complex, illustrating that all of these spin-triplet species can be coherently controlled. However, unlike the Cr^4+^ derivatives, these pseudo-tetrahedral V^3+^ and Mo^4+^ complexes exhibit no measurable emission. Coupling absorption spectroscopy with computational predictions, we investigate why these complexes exhibit no detectable photoluminescence. These cumulative results suggest that design of future V^3+^ complexes should target pseudo-tetrahedral symmetries using bidentate or tridentate ligand scaffolds, ideally with deuterated or fluorinated ligand environments. We also suggest that spin-triplet Mo^4+^, and by extension W^4+^, complexes may not be suitable candidate optically addressable qubit systems due to their low energy spin-singlet states. By understanding the failures and successes of these systems, we outline additional design features for optically addressable V- or Mo-based molecules to expand the library of tailor-made quantum sensors.

## Abstract 2

Enabling coherent spin-control in integer spin complexes with molecular color center design.

## Graphical abstract

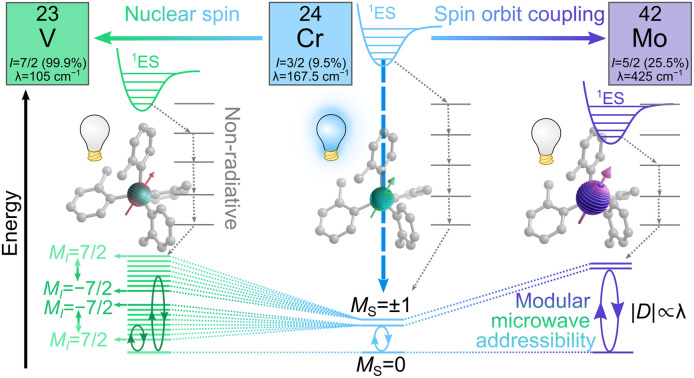

## Introduction

The second quantum revolution is driven by the design, creation, and control of quantum bits, or qubits, the fundamental units of quantum information processing.^1–10^ Harnessing the power of quantum control, quantum sensors have provided unprecedented visualizations of nanoscale magnetic and electric fields in diverse physical environments.^11–14^ In many impressive demonstrations – such as magnetic resonance of individual proteins,^12^ detection of action potentials of individual neurons,^15^ or mapping electron flow in two-dimensional materials^16,17^ – optically active, paramagnetic defects embedded in a crystalline host serve as the qubits.^3,7,18–22^ The electronic structure of these color centers provides a valuable platform for quantum control. First, the ground state spin sublevels of these defects provide a two-level quantum system to act as the qubit. Crucially, the spin sublevel populations may be optically prepared into a non-thermal equilibrium state, *i.e.* initialized, while the spin state information may be read out using spin-dependent optical emission.^18,23^ These combined features enable remote optical control and single qubit addressability, both of which are valuable features for quantum sensing technologies.^24,25^

Although solid-state color centers offer incredible coherence properties and quantum control, deterministic spin localization and defect tunability remain major challenges.^7^ To complement existing top-down creation of these solid-state systems, molecular synthesis provides a bottom-up approach, offering a modular and scalable route to develop emerging quantum technologies.^5,7,26–31^ To create bespoke quantum sensors, we sought to develop molecular analogues of solid-state color centers that host a similar optical-spin interface, but with their molecular nature facilitating bottom-up design and solution processibility towards specific sensor–analyte interactions.^32,33^ In recapitulating this optical-spin interface, we needed (i) a ground state spin that can be coherently controlled, (ii) an electronic excited state that is ‘connected’ to this ground-state spin through a spin-selective optical process (*e.g.*, excitation or relaxation, see ESI† for further discussion), (iii) radiative decay from the excited state to the ground state for optical readout, and (iv) a ground-state spin lifetime, or spin-lattice relaxation time (*T*_1_), that is longer than the optical lifetime (*τ*_opt_).^34^ These criteria initially led us to spin-triplet (*S* = 1) transition metal complexes that share a similar electronic structure with optically addressable solid-state spins such as the anionic nitrogen vacancy center in diamond, divacancy defects in silicon carbide, or more similarly, tetravalent chromium (Cr^4+^) dopants in silicon carbide.^35–38^

While these generic features provide a framework to build molecular color centers, *S* = 1 molecular systems pose two key challenges for spin-dependent optical readout. First, to achieve a spin-selective optical process and emission in *S* = 1 complexes, a ligand field that ensures that the first excited state is a spin-singlet excited state (^1^ES) is desirable.^39^ For resonant optical control (off-resonant optical control protocol addressed in the ESI†), the spin-flip transition from the ^1^ES to the spin-triplet ground state (^3^GS) should exhibit limited vibronic coupling, allowing for both narrow optical linewidths for spin-selective optical excitation and radiative decay to the ground state.^34,40^ This desired electronic structure requires a sufficiently strong ligand field around the spin-bearing metal center such that the first excited state is a ^1^ES, eliminating lower energy ^3^ES's that provide non-radiative decay pathways.^39^ Second, the ground-state spin must be capable of coherent control. However, the spin of *S* = 1 transition metal systems often cannot be coherently controlled with readily accessible microwave frequencies (*i.e.*, 1–40 GHz). The inability to coherent drive the ground-state spin of these systems arises from both the integer spin and large zero-field splitting (ZFS) values, requiring high microwave frequencies (>95 GHz) to probe ground-state spin transitions.^41,42^ This challenge may be overcome by designing orbitally non-degenerate, *S* = 1 ground states, wherein the spin-bearing ion resides in a (near) cubic ligand field.

Considering these design parameters, we previously demonstrated that pseudo-tetrahedral (*T*_d_) Cr^4+^ molecules in a strong ligand field exhibit small ground-state ZFS values of 1.8–4.1 GHz for microwave control at X-band frequency (9–10 GHz), and an optical-spin interface.^34,40^ Yet, the air-sensitivity, small spin–orbit coupling, and low percentage of nuclear spin-bearing isotopes of Cr^4+^-based systems may not be optimal for every sensing task. Herein, we aim to translate this combination of features from *T*_d_ Cr^4+^ systems into molecular hosts that offer (i) intrinsic nuclear spins, *I*, to serve as quantum memories for prolonged information storage,^7,43,44^ and (ii) larger spin–orbit coupling for improved sensitivity to electric fields.^35,45^ To that end, we turned to *T*_d_ trivalent vanadium (V^3+^) and tetravalent molybdenum (Mo^4+^) derivatives in the same ligand fields as their Cr^4+^ congeners. The vanadium-51 (*i.e.*, ^51^V, *I* = 7/2, 99.8% abundance) center provides a potential nuclear spin memory that is intrinsically coupled to the electronic spin through hyperfine interactions,^42,46^ while the Mo^4+^ center increases the spin–orbit coupling experienced by the electron spin, offering enhanced sensitivity of the spin Hamiltonian parameters to certain external fields through the Zeeman splitting term (*e.g.*, strain or electric fields).^47,48^ However, the metal ion substitution should substantially change both the electronic structure and spin dynamics that dictate the quantum sensor performance. To initiate our studies with these ions, we synthesized and evaluated three spin-triplet systems with V^3+^ and Mo^4+^ to compare with previously studied Cr^4+^ congeners. Coupling spectroscopic analysis with computational predictions, we evaluate how to design these systems, addressing their advantages and limitations. From these results, we suggest that reducing nearby high energy oscillators and using ligand scaffolds could enable both emission and low frequency microwave control for V^3+^ systems while Mo^4+^ systems may be fundamentally limited by their intrinsically low lying ^1^ES.

## Results and discussion

**Fig. 1:**
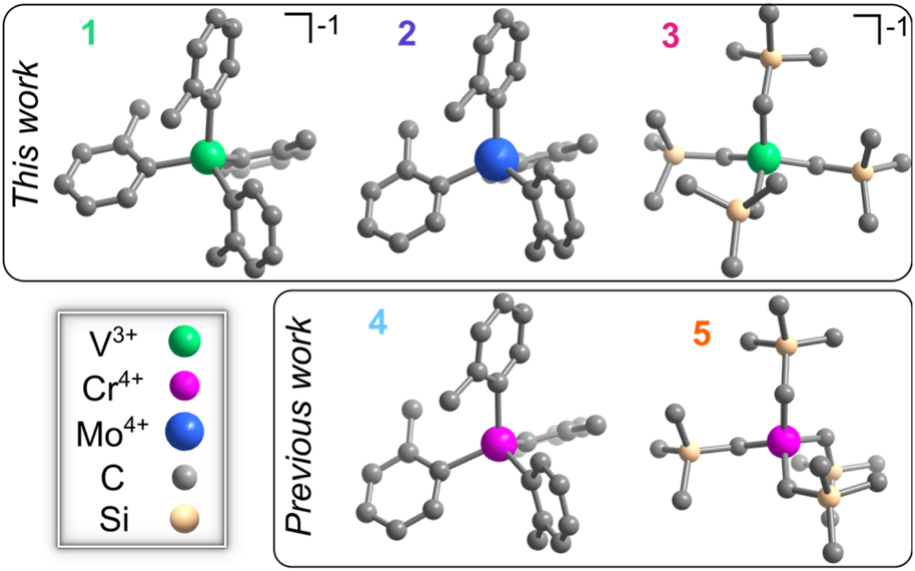
Molecular structures of 1, 2, 3, 4 (ref. 34), and 5 (ref. 40) determined at 100 K. Carbon, silicon, vanadium, chromium, and molybdenum given in gray, peach, green, pink, and blue, respectively. Hydrogen atoms and counter ions omitted for clarity.

We synthesized [Li(THF)_4_][V(*o*-tolyl)_4_] (1, THF = tetrahydrofuran), Mo(*o*-tolyl)_4_ (2),^49^ and [Li(12-crown-4)_2_][V(trimethylsilylmethyl)_4_] (3) to directly compare with previous candidate color centers,^34,40^ Cr(*o*-tolyl)_4_ (4)^50^ and Cr(trimethylsilylmethyl)_4_ (5)^51^ (Fig. 1). Detailed synthetic procedures for 1 and 3 are outlined in the ESI† while 2, 4, and 5 were synthesized according to previously reported methods.^49–51^ We also note that previous efforts to synthesize the molybdenum analogue of Cr(trimethylsilylmethyl)_4_ resulted in the hexakis(trimethylsilylmethyl)dimolybdenum species, so we could not investigate the Mo^4+^ analogue of 5.^52,53^

We first characterized the solid-state structure of 1 and 3 through single crystal X-ray diffraction, finding that their inner MC_4_ coordination sphere remains close to ideal tetrahedral symmetry based on both the *τ*_4_ and *τ*_4′_ metrics (0.95–1.01).^54,55^2 had been previously characterized at room temperature and shows only a slight contraction in Mo–C bond lengths upon cooling to 100 K.^49^ As such, these systems should exhibit relatively small, but non-zero, ZFS values. The ground-state structure may be approximated as a descent in symmetry from ideal *T*_d_ to *C*_2v_, as discussed for [Li(THF)_4_][V(mesityl)_4_] and [Li(THF)_4_] [V(pentachlorophenyl)_4_].^56^

**Fig. 2:**
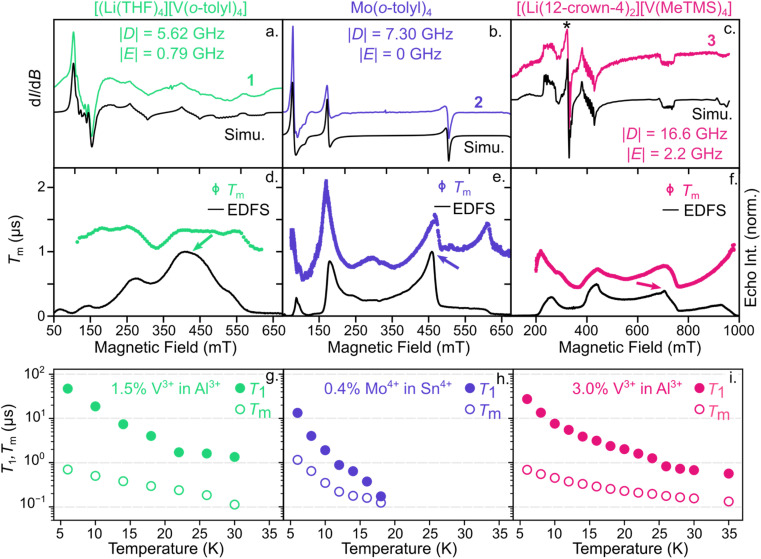
(top) cw-EPR spectra of (a) 1′, (b) 2′, and (c) 3′ at 5 K and 9.37–9.39 GHz with resulting *D* and *E* values. * denotes V^4+^ impurity in 3′, representing 1% of spin density in the sample. (middle) Magnetic field dependence of *T*_m_ (hollow symbols) at 6 K for (d) 1′, (e) 2′, and (f) 3 overlayed on the echo-detected field swept spectrum. Error bars are within the data points. (bottom) Temperature dependence of *T*_1_ (filled circles) and *T*_m_ (hollow circles) for (g) 1′, (h) 2′, and (i) 3′ at X-band frequency and measured at the magnetic field indicated by the arrow in the (d–f).

Microwave addressability remains a challenging parameter to design and tune in *S* = 1 compounds.^41,42^ Thus, we initiated our investigation by probing the ground-state spin structure of these systems to determine if they are capable of ground-state spin control. We characterized 1–3 with both continuous-wave (cw) and pulsed electron paramagnetic resonance (EPR) to understand their ground-state spin structure and dynamics. To reduce decoherence from electron spin-electron spin interactions, we cocrystallized 1–3 in their corresponding isostructural, diamagnetic analogues, [Li(THF)_4_][Al(*o*-tolyl)_4_] (1-Al), Sn(*o*-tolyl)_4_ (2-Sn),^57^ and [Li(12-crown-4)_2_][Al(trimethylsilylmethyl)_4_] (3-Al), respectively (see ESI† for details). We denote the cocrystallized samples as 1′–3′ for all subsequent experiments with relative electron spin concentrations of 0.36–3% (Table S8†). From the X-band cw-EPR spectra in Fig. 2a–c, we find that the axial ZFS values, *D*, are larger for the V^3+^ and Mo^4+^ derivatives than the corresponding Cr^4+^ congeners.^40^1′ and 2′ exhibit |*D*| values of 5.62 GHz and 7.3 GHz, respectively, while |*D*| for 4′ at 5 K is 3.63 GHz.^34^ The largest contribution of the two-fold increase in |*D*| for 2′ likely arises from the increased spin–orbit coupling of the heavier Mo^4+^ ion, where the free ion spin–orbit coupling parameter, *λ*, is ∼425 and 167.5 cm^−1^ for Mo^4+^ and Cr^4+^.^58^ Given the similarity in *λ* for V^3+^ and Cr^4+^, the increase in |*D*| for 1′ more likely results from symmetry lowering around the V^3+^ center due to crystal packing with the [Li(THF)_4_]^+^ cation. For example, |*D*| = 5.62 GHz for 1′ is similar to the |*D*| value of 5.55 GHz for Cr(*o*-tolyl)_4_ (4) diluted in a lower symmetry (orthorhombic) host matrix.^59^

While 1′ and 2′ show modest increases in |*D*| relative to their Cr^4+^ analogue (4′), 3′ exhibits a dramatic increase to 16.6 GHz, which is ∼13 times greater than its Cr^4+^ analogue (5′) where |*D*| = 1.23 GHz.^40^ To understand this order-of-magnitude increase in |*D*| for 3′, we quantified the deviation from ideal *T*_d_ symmetry using the *τ*_4_ and *τ*_4′_ parameters^54,55^ for 3′ and 5′. Between 3′ and 5′, the *τ*_4_ and *τ*_4′_ values vary by only 0.004 and 0.015, respectively. While these geometric deviations are small, the absolute spin splitting energies of 3′ and 5′ vary by only 63 μeV, suggesting that subtle structural changes may result in significant variations in |*D*|. However, variations in |*D*| may also arise from a convolution of competing effects, such as changes in SOC, ligand field strength, symmetry-driven coupling to excited states, and electron delocalization.^60,61^ Thus, to maintain microwave addressability across diverse host media for sensing, it will be key to accurately predict and measure how growth on different surfaces or substrates affects local structure around the spin center and, consequently, |*D*|.^62^

The two V^3+^ complexes, 1′ and 3′, also exhibit two advantageous features. First, the crystal symmetries of 1′ and 3′ result in non-zero transverse ZFS, |*E*|, values of 0.8 and 2.2 GHz, respectively. Non-zero |*E*| should result in clock-like transitions around zero magnetic field at frequencies of |*D* − *E*|, |*D* + *E*| and 2|*E*|, resulting from mixing of the *M*_S_ = +1 and −1 states (Fig. S1†).^63^ Clock-like transitions show little variation in their transition frequency with small changes in applied magnetic field.^64,65^ As a result, the clock transitions at zero magnetic field should exhibit longer coherence times, *T*_m_, than typical Zeeman transitions, which exhibit a linear dependence on the external magnetic field.^30,59,64–67^ Second, the cw-EPR spectra in Fig. 2a and c exhibit hyperfine coupling to the ^51^V nucleus of 155 and 165 MHz for 1′ and 3′, respectively. Thus, the built-in ^51^V nuclear spin and relatively large hyperfine coupling to the electron spin provides a potential nuclear spin memory to prolong the storage time of quantum information.^44,68,69^ For example, previous work with *S* = 1/2 V^4+^ as well as ^173^Yb^3+^ molecular spins suggested that the coherence of strongly coupled nuclear spin may be prolonged beyond electronic spin coherence time by increasing the magnetic field strength.^46,70^ As a result, both the clock-like transitions around zero field and the intrinsic nuclear spin of the V^3+^-based systems provide potential routes to lengthen coherence lifetimes.

To then determine the spin dynamics of these Mo^4+^ and V^3+^ systems in their isostructural matrices, we examined the temperature and magnetic field dependence on spin-lattice relaxation (*T*_1_) and coherence (*T*_m_) times for 1′–3′ at X-band frequency (9–10 GHz). In each case, these complexes exhibit shorter *T*_1_ times (10–15 μs) at low temperature than 4′ or 5′, where we previously measured *T*_1_ times of 2–3 ms at 5 K.^40^ However, the concentration of both 1′ and 3′ were 1.5 and 3% in the spin-diluted lattice, which we have previously shown leads to a reduction in *T*_1_ at low temperatures for 4′.^40^ Thus, *T*_1_ for 1′ and 3′ may likely be improved with further dilution. Notably, 1′ and 3′ exhibit *T*_1_ times of >0.5 μs up to 30 K while 2′ shows a steep decline in *T*_1_ < 0.5 μs by 18 K (Fig. 2g–i). These contrasting temperature dependencies likely arise from the larger spin–orbit coupling of the Mo^4+^-based spin centers, as well as lower energy phonon modes arising from the heavier metal center.^71,72^

Turning to the *T*_m_ times, a key metric to evaluate the performance of qubit candidates, we find relatively similar temperature dependencies for 1′–3′. In general, each compound exhibits *T*_m_ times of approximately 1 μs at 6 K, likely limited by the high density of ^1^H nuclei on the ligands.^23,73,74^ To verify that the nuclear spin environment inhibits *T*_m_, we also performed power-dependent Hahn-echo experiments to mitigate the influence of instantaneous diffusion from nearby paramagnetic spin centers in relatively spin concentrated samples.^64,75^ We find that the *T*_m_ times of 1′ and 3′ show no significant change with decreasing power (Fig. S2†), suggesting that the nuclear spin environment of the matrix limits *T*_m_ in these samples. In contrast, the *T*_m_ time of 2′ approaches ∼2 μs at decreasing small microwave powers, which is similar to the *T*_m_ time previously measured for 4′ at similar concentrations^40^ (see ESI† for further details). This analysis does not result in large increases in *T*_m_ times, indicating that coherence times reported here are likely limited by the ^1^H nuclear spin environment. The *T*_m_ times then decrease with increasing temperature until the *T*_m_ times are limited by *T*_1_. The larger spin–orbit coupling in 2′ likely leads to *T*_1_-limited *T*_m_ times by 18 K while the V^3+^-based systems are measurable up to at least 30 K. However, both the *T*_1_ and *T*_m_ times in 1′ and 3′ are shorter than their Cr^4+^ congeners, 4′ and 5′.^40^ We posit that in these low field measurements, we may be either simultaneously driving multiple overlapping spin transitions (Fig. S1†) or driving a highly mixed spin transition, which has previously resulted in decreased spin-lattice relaxation times for an *S* = 5/2 Fe^3+^ complex.^76^ However, this assertion requires a multifrequency EPR study for validation, which will be the subject of future work. Additionally, we note that the *T*_1_ and *T*_m_ times reported here for 1′–3′ will vary substantially when measured in other matrices as the matrix tends to most strongly influence the spin dynamics of molecular qubits.^77^

We further evaluate the magnetic field dependence of these *T*_m_ times. Similar to the behavior of 4′,^40^ we find local maxima in *T*_m_ for 2′ at 170, 460, and 620 mT, where the applied field is parallel or perpendicular to the principal axis of the ZFS tensor (Fig. 2e). Across this field range, *T*_m_ changes by a factor of ∼4. For 1′ and 3′ where *E* > 0 and *I* = 7/2, the field dependence of *T*_m_ is less exaggerated since these systems exhibit are more transitions over a similar field range (Fig. S1†). As a result, *T*_m_ changes by less than a factor of 2 for 1′ over a similar field range to 2′. While the variation is less significant for 1′ and 3′, these data still highlight that no single value of *T*_m_ provides a complete picture of the spin relaxation, especially for anisotropic metal complexes. We similarly expect *T*_1_ to show a strong magnetic field dependence for 1′–3′, as demonstrated with Cr(*o*-tolyl)_4_.^78^

Most importantly, even the maxima in *T*_m_ times measured here still fall well short of coherence times for state-of-the-art quantum sensors, such as anionic nitrogen vacancy centers in diamond (*e.g.*, from 10 s to 100 s of microseconds).^79^ In fact, low temperature (≤10 K) *T*_m_ times across most transition metal-based systems to date are ≤15 μs, regardless of spin state,^30,73,77,80,81^ ligand nuclear spin environment,^77,82^ or magnetic field,^40,83^ illustrating how the surrounding matrix promotes decoherence. Thus, introducing clock-like transitions (*e.g.*, Fig. S1†) into molecular sensors while be critical to improve their coherence properties, and consequently their sensitivity,^84^ in magnetically noisy matrices.^23,63^

**Fig. 3:**
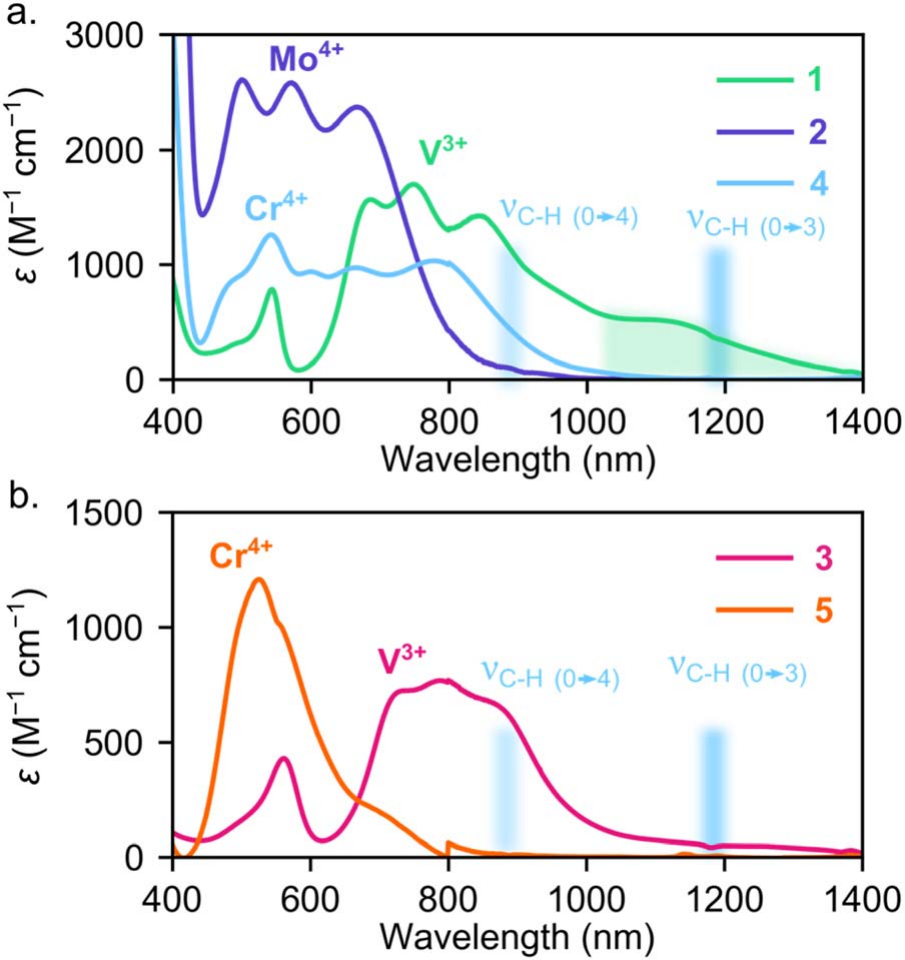
(a) Solution-state electronic absorption spectra of 1 and 2 compared with Cr^4+^ analogue, 4 (ref. 40). Green highlighted region indicates low energy absorption present for [V(aryl)_4_]^−^ anions. (b) Solution-state electronic absorption spectra of 3 compared with Cr^4+^ analogue, 5 (ref. 40). Blue shaded region highlights energy of C–H stretching overtones that can mediate non-radiative decay.

Turning to the excited state structure, we initially measured optical absorption spectra of these systems in solution at room temperature. The solution-phase UV-visible near-infrared spectra in Fig. 3a illustrate that the Mo^4+^ ion in 2 leads to both higher energy and more intense transitions than either of the first row congeners, 1 and 4. The UV-vis-NIR spectrum of 2 also appears qualitatively similar to 4, but the transitions in 2 are shifted to higher energy (Fig. S6†). This spectrum suggests that, similar to 4, the ^1^ES of 2 is the lowest energy excited state, which should yield the correct energy level structure for optical-spin control.^34^ Conversely, the V^3+^ ion in the pseudo-*T*_d_*o*-tolyl ligand field of 1 leads to lower energy transitions than both 2 or 4. In fact, 1 exhibits absorption extending well into the NIR, leading to substantial spectral overlap with aromatic C–H overtones (see green and blue shaded regions in Fig. 3a), which may provide a multi-phonon mediated non-radiative decay pathway.^85^

When replacing the aryl ligands in 1 with (trimethylsilyl)methyl ligands in 3, we find that the absorption features from 1100–1400 nm are suppressed while the shorter wavelength transitions from 400–1000 nm appear similar between 1 and 3. The reduction in absorption between 1100–1400 nm for 3 suggests that either the stronger alkyl ligand field increases the transition energy of the ^3^ES manifold or the σ-only alkyl ligands do not mix with the lowest energy ^3^ES metal-centered transitions, resulting in very low oscillator strengths of the d–d transitions. We observed similar behavior in tetraaryl- and tetralkyl-Cr^4+^ systems. For example, 4 exhibits intense absorption between 600 and 800 nm while those transitions are present, but much weaker, for 5.^40^

To determine if 1–3 exhibited emission from a ^1^ES, we then performed steady-state photoluminescence measurements. When exciting pure, single-crystalline samples of 1–3 at 4 K with 660 or 785 nm excitation, we observe no emission in the range of 900–1700 nm. Even performing photoluminescence experiments on dilute single-crystalline samples of 1′–3′ or films of 5–10% (w/w) of 1–3 in polystyrene at 4 K yielded no emission. The lack of emission prevents optical readout of the ground-state spin, the essential component for molecular color centers. We sought to understand why some systems emit and other similar ones did not, to see if failure might serve as a guide for future systems.

**Fig. 4:**
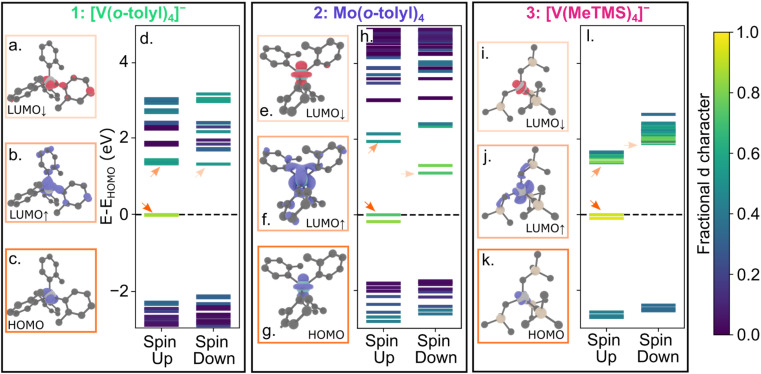
Visualizations of selected molecular orbitals for 1 (a–c), 2 (e–g), and 3 (i–k). The color of the box around each molecular orbital visualization corresponds to the quantitative molecular orbital diagram for 1 (d), 2 (h), and 3 (l). The LUMO_↑_ and LUMO_↓_ orbitals represent the first spin-triplet (^3^ES) and first spin-singlet (^1^ES) excited states, respectively.

We performed spin polarized density functional theory calculations using VASP (Vienna *Ab initio* Simulation Package) 6.3.2 (ref. 86–89) with projector augmented wave pseudopotentials^90,91^ and the PBE exchange correlation functional^92,93^ (see ESI† for further details). For calculations of the charged molecules 1 and 3, we explicitly included counterions but, for clarity, we only highlight spin-bearing orbitals here. Fig. 4 shows the electronic structures of 1–3, highlighting the d character of the molecular orbitals. We visualized the highest occupied molecular orbital (HOMO) and spin up and down lowest unoccupied molecular orbital (LUMO) orbitals. Similar to 4 and 5,^40^ all molecules have a HOMO and LUMO with significant d character. Thus, the qualitative picture of all five compounds is similar, yet only 4 and 5 exhibit measurable emission.

To evaluate where the ^1^ES lies relative to the ^3^GS manifold, we performed excited state calculations using the ΔSCF (delta self consistent field) method.^94,95^ We calculated the ^1^ES-^3^GS gap by subtracting the total energy of a constrained occupancy calculation, where the electron in the spin-up HOMO is promoted to the spin-down LUMO, from a standard ground-state DFT calculation (see ESI† for further details). We calculate ^1^ES-^3^GS gaps of 0.355 eV, 0.319 eV, and 0.407 eV, eV for 1, 2, and 3, respectively. Previously, we estimated the ^1^ES-^3^GS gap is 0.537 eV for 4 using the same methodology.^62^ Thus, the calculated ^1^ES energies for 1, 2, and 3 are only 66, 59, and 76% of 4. Although these values have limited quantitative accuracy, the trend suggests that the ^1^ES energy of 1 and 2 is significantly lower than 4. These results align with the expectation that the spin-pairing energetic penalty decreases with increasing ionic radii, such that the ^1^ES energies should be Mo^4+^ < V^3+^ < Cr^4+^ in the same ligand field. We further find that 3 also exhibits a lower calculated ^1^ES value than 4, despite the stronger alkyl ligand field of 3.

If we estimate hypothetical emission wavelengths by assuming the ratio between the calculated ^1^ES and experimental emission energies is similar to 4, where calculated ^1^ES/emission energy = 0.537/1.209 eV,^34,62^ we find that 1, 2, and 3 would emit at roughly 1550, 1800, and 1400 nm. Considering this wavelength range, the lack of measurable emission may therefore be rationalized by four possible effects. First, any photons emitted at longer wavelengths than 1700 nm are outside of the range of commercially available InGaAs NIR detectors that are optimal for typical NIR emission measurements. Second, if any emission occurs in the measurement window, the higher strain sensitivity of 1–3 with lower symmetry or larger spin orbit couplings could result in broader emission than Cr^4^ derivatives, resulting in fewer photons counted per pixel in the CCD measurements, rendering the measurement less sensitive. Third, the posited lower energy ^1^ES (≫1200 nm) may exhibit significant spectral overlap with high energy C–H stretching overtones (*e.g.*, Fig. 3) that provide highly favorable non-radiative decay through multi-phonon mediated relaxation pathways. This potential explanation for the lack of emission could be ameliorated through selective deuteration or fluorination of the ligands.^96^ Further experimental and theoretical studies are required to investigate this pathway. Fourth, the energy-gap law suggests that as the emissive state becomes lower in energy, non-radiative decay rates will increase.^97,98^ In each compound, a combination of these effects may be operative, leading to no detectable emission. Based on the literature and our studies of similar compounds, we hypothesize that the C–H overtones are critical for quenching emission in this energy region.^96,99–101^ These results suggest a pathway forward for the design of molecular color centers featuring depleted C–H modes for compounds that emit in the near-IR or telecom region.

### Designing emissive V^3+^ systems with a microwave addressable ground-state spin

The aggregate of these results provides us with a series of new *S* = 1 molecules featuring coherent control over their ground-state spin and a set of design principles for next generation quantum sensors. Previous data illustrate that emissive V^3+^ systems may be achieved using trigonal bipyramidal or octahedral geometries.^102,103^ However, trigonal bipyramidal or octahedral geometries with d^2^ metal ions result in either a non-cubic (*e.g.*, *C*_3v_, *D*_3h_) symmetry or an orbitally degenerate (^3^T_2_) ground state, respectively. These features result in |*D*| values that well exceed frequencies of conventional microwave sources, making their ground-state spin control more challenging. Thus, we attempted to unify the emissive nature of five and six coordinate V^3+^ with the low |*D*| values of pseudo-*T*_d_ by introducing the V^3+^ ion into sufficiently strong ligand fields. However, we never observed emission for V^3+^ systems, even with the strong-field alkyl donors of 3. These results suggest that a stronger ligand field should be enforced through a rigid ligand coordination with, for example, *C*_3_ symmetric ligand scaffolds (*e.g.* tris(pyridyl)methane, tris(*N*-heterocyclic carbene)borate) or *C*_2_-symmetric bidentate ligand scaffolds (*e.g.* 1,1′-binaphthyl-2,2′-diamine).^104^ Notably, increased rigidity is often ascribed to improved radiative efficiency of Cr^3+^ systems.^96^ However, the hard nature and high charge of early transition metals likely necessitates using suitably hard, anionic ligand donors, precluding the use of softer strong field ligands such as phosphines. Additionally, the lower energy transitions in V^3+^ systems likely undergo rapid non-radiative decay mediated by nearby C–H groups. Thus, the spectral overlap of these modes with excited states may be mitigated through deuteration or fluorination, also demonstrated with Cr^3+^ systems to greatly enhance emission.^96,99,100^ A similar approach may be employed with future systems to realize emission from these V^3+^ systems that feature generally small (*e.g.*, <30 GHz) |*D*| values.

### Designing emissive Mo/W systems with microwave addressable ground-state spin

In contrast to the V^3+^ analogues, Mo^4+^-based systems pose a unique challenge. The energy gap between the ^1^ES and ^3^GS is generally quite small given the large size of the Mo^4+^ center. Additionally, to unlock the full potential of large spin–orbit coupling from the Mo^4+^ center to achieve enhanced sensitivities of the spin to electric field perturbations, the ion should sit in a polar molecular symmetry, such as *C*_3v_.^45^ For Mo^4+^ systems, these symmetries may simply yield an *S* = 0 complexes or prohibitively large |*D*| values. Thus, for Mo- and even W-based quantum sensors, an alternative spin-state may be better suited to enable strong spin-electric field coupling.^47,105^ For example, Mo^5+^*S* = 1/2 defects in silicon carbide have demonstrated NIR emission^106^ and may be capable of coherent optical control, similar to anionic tin vacancies in diamond.^107^ Thus, future investigations with *S* = 1/2 Mo^5+^/W^5+^ systems may provide a more suitable framework to achieve a ground-state spin capable of coherent control connected to an emissive optical state than the *S* = 1 derivatives with second or third row metal ions.

## Conclusions

In this work, we examined the electronic structure and spin dynamics of three novel V^3+^ and Mo^4+^ molecular color center candidates that could feature valuable spectroscopic handles for sensing. Notably, each system showed appropriately small ground-state anisotropy for coherent spin control. We demonstrated this control over a series of molecules, including a rare example of spin control in a second row transition metal with a spin-triplet ground state. We also suggest that the transverse anisotropy (*i.e.*, |*E*| > 0) and nuclear spin of the V^3+^ analogues may offer two avenues to extend quantum coherence. Despite these potential advantageous features, none of the systems investigated here exhibited measurable emission, likely resulting from a combination of a low energy ^1^ES and rapid non-radiative decay mediated by the C–H-rich ligand environment. Coupling experimental and computational insight, we suggest that the use of ligand scaffolds and deuteration/fluorination may result in emissive V^3+^ while maintaining ground-state spin control, while larger transition metal ions, such as Mo and W, in *S* = 1/2 states may be better suited for optical-spin control. Thus, we offer design considerations that will hopefully lead to the realization of V- or Mo-based molecular quantum sensors in future studies.

## Author contributions

D. W. L., L. R. W., S. L. B., P. D. performed the experimental measurements. K. R. M. performed the computations. D. W. L. synthesized the compounds. D. D. A., J. M. R. and D. E. F. advised on all efforts. All authors contributed to the data analysis and manuscript preparation.

## Conflicts of interest

The authors declare the following competing financial interests: D. W. L., S. L. B., D. E. F., and D. D. A. are inventors on patent application no. 63008589 submitted by the University of Chicago that covers chemically tunable optically addressable molecular-spin qubits and associated methods. S. L. B., P. D., D. W. L., D. E. F., and D. D. A. are inventors on patent application No. 22-T121 by the University of Chicago that covers enhancing quantum properties of optically addressable molecular qubits through host-matrix engineering.

## Supplementary Material

SC-015-D4SC03107E-s001

SC-015-D4SC03107E-s002

## Data Availability

The data underlying this work are available at https://doi.org/10.5281/zenodo.12794616. CCDC codes: 1 (2334908), 2 at 100 K (2334909), 3 (2334912), 1-Al (2334910), and 3-Al (2334911).
