# Supplementary material for: Coherent spin-control of S = 1 vanadium and molybdenum complexes
Source: Chem Sci. 2024 Aug 5;15(34):14016–26. doi: 10.1039/d4sc03107e (PMC11318652; doi:10.1039/d4sc03107e)
Supplement: SC-015-D4SC03107E-s001 [file SC-015-D4SC03107E-s001.pdf]

Supplementary Information (SI) for Chemical Science.
This journal is © The Royal Society of Chemistry 2024

Supplementary Information for:

Coherent spin-control of $S = 1$ vanadium and molybdenum complexes

Daniel W. Laorenza,[a,] Kathleen R. Mullin,[b] Leah R. Weiss,[c,d] Sam L. Bayliss,[c,e] Pratiti Deb,[c,f] David D. Awschalom,[*,c,f,g] James M. Rondinelli,[*,b] Danna E. Freedman[*,a]

a. Department of Chemistry, Massachusetts Institute of Technology, Cambridge, Massachusetts, 02139, USA.
b. Department of Materials Science and Engineering, Northwestern University, Evanston, Illinois, 60208, USA.
c. Pritzker School of Molecular Engineering, University of Chicago, Chicago, Illinois 60637, United States.
d. Advanced Institute for Materials Research (AIMR-WPI), Tohoku University, Sendai 980-8577, Japan
e. James Watt School of Engineering, University of Glasgow, Glasgow G12 8QQ, United Kingdom
f. Department of Physics, University of Chicago, Chicago, Illinois 60637, United States
g. Center for Molecular Engineering and Materials Science Division, Argonne National Laboratory, Lemont, Illinois 60439, United States

Full Experimental Details S3
Supplementary text S13
Tables S1-S5 | Summary of crystallographic data for **1-3**, **1-Al**, and **3-Al** at 100 K S16
Table S6 | Simulation parameters for the EPR spectra of **1′-3′** S21
Table S7 | Structural parameters and ZFS values for **1-3** S21
Table S8 | Qubit:host ratios determined from ICP-OES for **1′-3′** S21
Figures S1 | Example Zeeman splitting diagrams for **1′** S22
Figures S2 | Power-dependent pulsed EPR experiments for **1′-3′** S23
Figures S3-S5 | UV-Vis-NIR spectra and Beer-Lambert Law analysis for **1**-**3** S24
Figures S6 | Comparison of UV-Vis-NIR spectra for **2** and **4** S27
Figures S7 | Spectral deconvolution of UV-Vis-NIR spectra for **1-3** S28
Figures S8-S13 | Electrochemistry data for **1-3** in THF S29
Figures S14-S19 | NMR data for **1-Al** and **3-Al** S35
Figures S20-S22 | NMR data for **1** and **3** S41
References S44

Full Experimental Details

General considerations and synthesis. Glassware was either oven-dried at 150 °C for at least four hours or flame-dried prior to use. Tetrahydrofuran (THF), diethylether (Et_2O), and hexanes were dried using a commercial solvent purification system from Pure Process Technology and stored over 4 Å sieves prior to use. These solvents were subjected to a test with a standard purple solution of sodium benzophenone ketyl in THF to confirm low O_2 and H_2O content prior to use. Aluminum trichloride, 1,4-dioxane (anhydrous), (trimethylsilyl)methylmagnesium chloride (1.0 M in diethyl ether), *o*-tolylmagnesium bromide (2.0 M in diethyl ether), and *n*-butyllithium (2.5 M in hexanes) were purchased from Sigma Aldrich and used as received. (Trimethylsilyl)methyllithium solution (10 wt% in hexanes, ~0.7 M) was purchased from Acros Organics and used as received. 2-bromotoluene was purchased from Sigma Aldrich and subjected to three freeze-pump-thaw cycles followed by drying for at least 24 hours over 4 Å sieves prior to use. $VCl_3\cdot3(THF)$,[1] *o*-tolyllithium,[2] and $MoCl_4\cdot2(Et_2O)$[3] were prepared according to literature preparations. Both *o*-tolyllitihum and $MoCl_4\cdot2(Et_2O)$ were stored at −35°C prior to use. Typically, $MoCl_4\cdot2(Et_2O)$ was freshly prepared within 48 hours of use as the material is not indefinitely stable, even at −35°C.

Synthetic procedures. All experimental manipulations were performed in an N_2 atmosphere with either an MBraun Unilab Pro glovebox, Vacuum Atmosphere Nexus II glovebox, or Schlenk techniques unless otherwise explicitly stated. The purity of vanadium compounds was determined through a combination of NMR and EPR due to challenges with combustion analysis of air-sensitive organometallic reagents. See procedures described below for details.

[Li(THF)$_4$][V(*o*-tolyl)$_4$] (1).

Step 1: In-situ preparation of V(*o*-tolyl)$_3$THF. 373 mg of $VCl_3\cdot3(THF)$ (1 mmol, 1 equiv.) was suspended in 10 mL of THF in a 20 mL scintillation vial. At room temperature, 1.6 mL of 2.0 M *o*-tolylmagnesium bromide in Et_2O (3.2 mmol, 3.2 equiv.) were added dropwise to the stirring slurry of $VCl_3\cdot3(THF)$ in THF over ~1 minute. The reaction mixture turned a dark blue immediately following the addition and retained the deep blue color for the next 90 min of stirring. [Note 1: If the addition is done on a larger scale, the reaction should be cooled to avoid heating. However, the reaction mixture will appear brownish immediately after addition at low temperatures and will not turn deep blue until the reaction flask has warmed to room temperature.]

After 90 min, 1 mL of 1,4-dioxane was added to precipitate out a white magnesium-halide-dioxane adduct. [Note 2: This precipitate is incredibly challenging to remove via filtration through filter paper or fritted funnels. Centrifugation is a much cleaner, faster alternative.] The cap of the scintillation vial was then electrical taped. [Note 3: Wrapping electrical tape at least three times around the vial cap/vial usually creates a sufficient seal to maintain a clean atmosphere for 15-20 minutes outside of the glovebox.] The vial was removed from the glovebox and immediately placed in a centrifuge and centrifuged for ~10 min. After this process, the white solid should be clearly separated from the deep blue supernatant [Note 4: If the solution appears brown, the V(*o*-tolyl)$_3$THF product has likely degraded from either exposure to oxygen from an imperfect seal or high water/peroxide contaminant content in the 1,4-dioxane]. The vial was then loaded back into the glovebox. The deep blue solution was then carefully decanted from the white solid. This blue solution contains the desired V(*o*-tolyl)$_3$THF product which was used without further purification or isolation in the next step.

Step 2: Preparation of [Li(THF)$_4$][V(*o*-tolyl)$_4$] from V(*o*-tolyl)$_3$THF. The vial containing V(*o*-tolyl)$_3$THF as prepared above was cooled to −30°C. Then, 110 mg of *o*-tolyllithium (1.12 mmol, 1.12 equiv. assuming 100% yield of V(otolyl)$_3$THF) was dissolved in 2 mL of Et_2O was cooled to −30°C. After 5-10 min, the *o*-tolyllithium solution was added dropwise over ~1 min to the THF solution of V(otolyl)$_3$THF. The reaction was stirred at −30°C for 30 min, then removed from the cold bath and stirred for 1 hour at room temperature. [Note 4: After taking the vial out of the cold well, the color changed from a blueish-purple to more of a brownish-purple over the course of ~20 minutes]. The solution was then filtered through a pad of celite and concentrated under vacuum to ~5-7 mL. The resulting solution was layered under 10 mL of Et_2O and stored at −30°C overnight. This procedure typically yielded single-crystal quality deep purple crystals within 16-24 hours. Total yield of crystalline product was generally 35±10% (~250 mg) based on VCl_3·3(THF) starting material. [Note 5: The crystals are dark purple and appear brown under an optical microscope in oil. The crystals are plate-like and often grow twinned with two plates joined together.]

Given the challenge of reliably performing elemental analysis on these air-sensitive, organometallic samples, we assessed the bulk purity of the product using a combination of ^{1}H NMR and cw-EPR to determine the presence of diamagnetic and paramagnetic impurities, respectively. Figure S20 and S21 show the ^{1}H NMR spectrum for [Li(THF)$_4$][V(*o*-tolyl)$_4$] in C_6D_6 and THD-h$_8$. The ^{1}H NMR spectrum in C_6D_6 shows common NMR solvent impurities from THF

and grease[4] but show no evidence of unreacted ligand or ligand decomposition. Similarly, the ^1H NMR spectrum in THF-h_8 shows no peaks from diamagnetic species, suggesting that the only impurities present may arise from paramagnetic species. From the cw-EPR spectrum of $[Li(THF)_4][V(o\text{-tolyl})_4]$, we see no evidence of other paramagnetic products around g ≈ 2 ± 0.2, suggesting the only paramagnetic species present is the S = 1 product. These cumulative results suggested that the product was pure but cannot definitively rule out other impurities that are neither NMR nor EPR active.

^1H NMR (400 MHz, THF-h_8): δ = 11 (br, s), 19.25 (br, s). ^1H NMR (400 MHz, C_6D_6): δ = 3.1 (br, s), 11 (br, s), 20.32 (br, s). Cyclic voltammetry: $V^{(4+/3+)}$(vs. $Fc^{*(+/0)}$ in THF with 0.1 M tetrabutylammonium hexafluorophosphate) = −0.69 V. UV-Vis-NIR (room temperature, THF, ε given in parentheses): 1110 nm (510±50), 845 nm (1430±50), 749 nm (1710±50), 685 nm (1580±50), 544 nm (790±20).

Mo(*o*-tolyl)$_4$ (2). This compound was prepared according to a slight modification from the literature preparation. $MoCl_4{\cdot}2(Et_2O)$ was used instead of $MoCl_4{\cdot}2(THF)$ as the starting material molybdenum source Briefly, a solution of ~500 mg (~5.1 mmol) of *o*-tolyllithium in 5 mL of Et_2O was cooled to −30°C. At the same time, a mixture of $MoCl_4{\cdot}2(Et_2O)$ in 10 mL of Et_2O was cooled to −30°C. After about 10 min, the solution of *o*-tolyllithium was slowly added over about 1-2 min to the stirring mixture of $MoCl_4{\cdot}2(Et_2O)$ in Et_2O. The reaction mixture was stirred in the cold bath for about an hour and then allowed to stir for at least 2 hours at room temperature. The resulting solution appeared as a deep bluish-purple. The product, **2**, was then isolated by filtering the solution through a pad of Celite and removing all volatiles under vacuum. Then the purple residue was extracted into hexanes at room temperature, filtered through a pad of Celite, and the solution was then concentrated to about 2-4 mL. Single crystals were grown from a concentrated hexanes solution at −35°C. The total reaction yields were similar (10-15%) to the previous report. For the purest material, **2** required recrystallization 2-3 times as follows: (*i*) the deep purplish-blue crystals were redissolved in minimal hexanes. (*ii*) The blue solution was filtered through a pad of celite and (*iii*) concentrated under vacuum. (*iv*) The solution was placed in the freezer −35°C and crystals generally grew within 16 hours. The process was repeated until no brown-ish precipitate was observed on the celite pad in step (*ii*).

^{1}H NMR (400 MHz, THF-h_8): δ = 42.6 (br, s), 51.9 (br, s), 91.7 (br, s). ^{1}H NMR (400 MHz, C_6D_6): δ = −112 (br, s), 42.4 (br, s), 50.8 (br, s), 91.6 (br, s). Cyclic voltammetry: $Mo^{(4+/3+)}$(vs. $Fc^{*(+/0)}$ in THF with 0.1 M tetrabutylammonium hexafluorophosphate) = −1.475 V. UV-Vis-NIR (room temperature, THF, ε given in parentheses): 668 nm (2440±80), 571 nm (2640±70), 500 nm (2650±60), 398 nm (10200±300).

[Li(12-crown-4)$_2$][V(MeTMS)$_4$] (3).

This compound could be prepared through two separate routes described below. The '*in situ* preparation' was generally higher yielding. Also, note that adding additional equivalents of the Grignard reagent in the first step does not result in the desired tetraalkyl V^{3+} product.

***In-situ* preparation.**

A mixture of 377 mg of $VCl_3\cdot3(THF)$ (1 mmol) in 10 mL of THF was cooled down to −30°C. Then, ~4 mL of 1 mM trimethylsilylmethylmagnesium chloride in Et_2O (~4 equiv relative to V) was added to solution in a single addition over about 1 min. The resulting brown solution was then removed from the cold bath and warmed up to room temperature over the next two hours, during which time the solution became a deep blue color. While the solution was stirring, 1-2 mL of anhydrous dioxane was then added into the solution. A fine white precipitate formed immediately and the solution was allowed to stir for 5 additional min (see Note 2 above). The cap of the scintillation vial was then electrical taped (see Note 3 above). The vial was removed from the glovebox and immediately placed in a centrifuge and centrifuged for ~10 min. After this process, the white solid was well separated from the deep purple supernatant. The vial was brought back into the nitrogen-filled glovebox and the purple supernatant was decanted from the white solid. The purple solution was then cooled back down to −30°C. Then, 1.43 mL of ~0.7 M trimethylsilylmethyl lithium in hexanes (1 mmol, 1 equiv) was added in a single addition to the vial. The solution was stirred at −30°C for 10 min., removed from the cold bath and stirred for 2 hrs. at room temperature. The THF solution was then concentrated to a purple sludge, at which point about 5-7 mL of Et_2O was added to create a uniform solution. With stirring, 704 mg (4 mmol, 4 equiv, 2-fold excess) of 12-crown-4 was added to solution. This solution was then concentrated to a out 5 mL and layered under 10-12 mL of hexanes and cooled to −35°C for 24 hours, during which time purple X-ray quality crystals grew. [Safety Note: These crystals should be handled carefully as they are pyrophoric and can ignite when exposed directly to air. All manipulations for X-ray analysis were performed under a nitrogen atmosphere or using oil to coat the crystals while

working in air.]. This solid was then redissolved in Et_2O, filtered through a pad of celite, concentrated to about 5 mL and then layered under 10-12 mL of hexanes again. After cooling to −35°C for 24 hours, purple crystals (572 mg, 72% yield) grew that were of better purity for EPR analyses. Typical yields following this method were 60-70%.

Stepwise procedure.

Step 1. Preparation of V(MeTMS)$_3$THF: A mixture of 260 mg of VCl_3·3(THF) (0.697 mmol) in 10 mL of THF was cooled down to −30°C. Then, ~2.8-2.9 mL of 1 mM trimethylsilylmethylmagnesium chloride in Et_2O (~4 equiv relative to V) was added to solution in a single addition over about 1 min. The resulting brown solution was then removed from the cold bath and warmed up to room temperature over the next two hours, during which time the solution became a deep blue color. While the solution was stirring, 1-2 mL of anhydrous dioxane was then added into the solution. A fine white precipitate formed immediately and the solution was allowed to stir for 5 additional min (see Note 2 above). The cap of the scintillation vial was then electrical taped (see Note 3 above). The vial was removed from the glovebox and immediately placed in a centrifuge and centrifuged for ~10 min. After this process, the white solid was well separated from the deep purple supernatant. The vial was brought back into the nitrogen-filled glovebox and the purple supernatant was decanted from the white solid. The solution was concentrated until a purplish sludge formed. This sludge was then triturated with hexanes (~15-20 mL) so that a solid purple product settled to the bottom of the vial. The supernatant was then decanted off and the solid was dried under vacuum. This product is poorly defined but has been previously described as the V(MeTMS)$_3$THF adduct, though its high solubility in THF has prevented structural assignment. Assuming the molar mass of V(MeTMS)$_3$THF, 150 mg (63% crude yield) of the deep purple solid was isolated. This yield could be increased by instead placing the hexanes/THF mixture in the freezer at −35°C for 16+ hours. The purple product is used as is without purification for the next step.

Step 2: Preparation of [Li(12-crown-4)$_2$][V(MeTMS)$_4$] from V(MeTMS)$_3$THF. 100 mg (0.21 mmol assuming formula unit of V(MeTMS)$_3$THF) of purple solid, 'V(MeTMS)$_3$THF', was dissolved in 5 mL of THF and cooled to −30°C. Then, 0.5 mL of ~0.7 M trimethylsilylmethyl lithium in hexanes (0.35 mmol, 1.66 equiv) was added to the stirring solution. The solution was stirred at −30°C, removed from the cold bath and stirred for 2 hrs at room temperature. After 2 hr, the solvent was removed under vacuum until only a purple sludge remained. Approximately, 1 mL

of THF, 2 mL of Et_2O and 5 mL of hexanes was added to the sludge to obtain to a cloudy mixture. After adding an additional 3 mL of THF to solubilize precipitates, 2.2 equiv of 12-crown-4 (110 mg, relative to V precursor) was added. During this time, a white-ish solid began to precipitate. The white solid was removed via filtration through a pad of celite. To the now purple solution, an additional 150 mg of 12-crown-4 was added, followed by 30 mL of hexanes. Then, a purple-ish solid/oil precipitated out along with a whiteish solid. The supernatant was decanted off and took the purple and white precipitates were taken back up in Et_2O. The white solid was insoluble in Et_2O and could be removed by filtration through a pad of celite. Additionally, the supernatant was cooled to −30°C for an hour, during which time additional purple solid precipitated. The supernatant was decanted again and the purple solid was redissolved in Et_2O. The resulting purple solution was filtered through a pad of celite and then combined with the purple solution obtained from the initial purple precipitate. The solvent was removed under vacuum to obtain about 45 mg, or ~20% yield. This method generally resulted in lower yields (~15-30%) than the '*in situ*' preparation outlined above.

As mentioned above for **1**, we assessed the bulk purity of the product using a combination of ^1H NMR and cw-EPR to determine the presence of diamagnetic and paramagnetic impurities, respectively. The ^1H NMR spectrum for [Li(12-crown-4)$_2$][V(MeTMS)$_4$] in THD-h$_8$ (Figure S22) shows no peaks from diamagnetic species, suggesting that the only impurities present may arise from paramagnetic species. Additionally, the diamagnetic peaks of the [Li(12-crown-4)$_2$]$^+$ likely overlap with the THF solvent peaks, obscuring those signals in the measurement. Attempts to perform ^1H NMR experiments in deuterated solvents were stymied by the insolubility of the complex in NMR solvents such as $CDCl_3$, CD_2Cl_2, C_6D_6, toluene-d$_8$ and the slow degradation of the product in more polar deuterated solvents (DMSO-d$_6$ and DMF-d$_7$). The cw-EPR spectra of [Li(12-crown-4)$_2$][V(MeTMS)$_4$] shows the presence of an $S = ½$ species that we posit results from ~1% of oxidized V^{4+} in the sample. This species was present across multiple samples. Given the propensity of [Li(12-crown-4)$_2$][V(MeTMS)$_4$] to oxidize, the impurity may arise from the bulk sample or may form during oxygen contamination during the EPR sample preparation stage. Continuous recrystallization from Et_2O/hexane mixtures helped to reduce, but not entirely eliminate, the presence of this impurity signal in the cw-EPR spectra. Note that powder samples of [Li(12-crown-4)$_2$][V(MeTMS)$_4$] should be handled with care when exposing them to moisture or oxygen as the product will rapidly decompose to a black tar (also see Safety Note above).

^{1}H NMR (400 MHz, THF-h_8) δ = −0.60 ppm (s, br, 183 Hz). Cyclic voltammetry: $V^{(4+/3+)}$(vs. $Fc^{*(+/0)}$ in THF with 0.1 M tetrabutylammonium hexafluorophosphate) −1.13 V. UV-Vis-NIR (room temperature, THF, ε given in parentheses): 870 nm (670±70), 787 nm (770±70), 728 nm (730±70), 561 nm (430±60).

[Li(THF)$_4$][Al(*o*-tolyl)$_4$] (1-Al). Tris(*o*-tolyl)aluminum has previously been prepared by reacting *o*-tolyllithium with $AlCl_3$ in THF.[5] Following this preparation, we purified the tris(*o*-tolyl)aluminum product from the LiCl byproduct by removing all THF and then redissolving the Al(*o*-tolyl)$_3$ in toluene and filtering off the LiCl salts. After removing the toluene, the subsequent Al(*o*-tolyl)$_3$ product was then used directly without further purification. Al(*o*-tolyl)$_3$ (~100 mg, 0.33 mmol) was dissolved in 2 mL of THF. In a separate vial, 50 mg (~0.5 mmol, ~1.5 equiv) of *o*-tolyllithium was dissolved in 4 mL of Et_2O. The Al(*o*-tolyl)$_3$ solution was then added to the stirring solution of *o*-tolyllithium at room temperature. The reaction proceeded for 2 hours and the solvent was then concentrated to roughly 2-3 mL. This reaction solution was layered under 7 mL of Et_2O to precipitate out microcrystalline [Li(THF)$_4$(Al(*o*-tolyl)$_4$] after 24 hours. This product was isolated by decanting off the supernatant and drying under vacuum. Then, the product was once again dissolved in 2 mL of THF and layered under 5 mL of Et_2O. After 2 days, single crystals suitable for single crystal X-ray diffraction grew. Typical reaction yields were about 30-40% based on the $AlCl_3$ starting material. ^{1}H NMR (400 MHz, $CDCl_3$) δ = 7.78 (dd, *J* = 7.0, 1.7 Hz, 1H), 7.05 (m, 3H), 3.53 (t, 4H), 2.29 (s, 3H), 1.80 (quin, 4H) ppm. ^{13}C NMR (101 MHz, $CDCl_3$) δ 147.65, 139.06, 128.45, 126.09, 123.82, 68.32, 25.57, 25.42 ppm. ^{27}Al NMR (104 MHz, THF) δ 135.38 ppm.

Sn(*o*-tolyl)$_4$ (2-Sn). This compound was synthesized according to previously published procedures.[6]

[Li(12-crown-4)$_2$][Al(MeTMS)$_4$] (3-Al).

150 mg of $AlCl_3$ (1.125 mmol) was dissolved in 10 mL of pre-cooled Et_2O at −30°C. This solution was removed from the cold well and then 8 mL of 0.7 M trimethylsilylmethyl lithium in hexanes (5.6 mmol, 5 equiv) was added to the stirred solution over about 5 minutes. After 1 hr, a white sludge started to form on the sides of the scintillation vial. At this point, ~650 mg (~4 mmol or ~3.5 equiv relative to Al) of 12-crown-4 was added in a single addition. The mixture was stirred for 16-18 hours. The resulting mixture was then filtered through a celite pad and volatiles were removed under vacuum to isolate a brown oily residue and white solid. This mixture was

redissolved in about 3 mL of Et_2O and layered under 7 mL of hexanes. After 24 hours at room temperature, the resulting white solid was isolated. Average reaction yields were about 20-30%. NMR in d6-benzene (contaminated with THF, benzene and toluene): ^{1}H NMR (400 MHz, $CDCl_3$) δ 3.71 (s, 4H), −0.07 (s, 5H), −1.24 (d, J = 27.2 Hz, 1H) ppm. ^{13}C NMR (101 MHz, $CDCl_3$) δ 66.18, 3.92 ppm. ^{27}Al NMR (104 MHz, THF) δ 150.952 ppm.

Electron Paramagnetic Resonance Measurements.

Preparation.

In a nitrogen glovebox, **1**, **2**, or **3** and analogous diamagnetic analogues, **1-Al**, **2-Sn**, **3-Al**, respectively were dissolved in ~1:100 w/w ratio in THF (or Et_2O for **3**/**3-Al**). The total mass of The THF (or Et_2O) solution was then concentrated under vacuum until the solution was nearly saturated. This THF (or Et_2O) solution was then layered under Et_2O (or hexanes) in approximately a 1:1.5 v/v ratio of THF:Et_2O (or Et_2O/hexanes). Crystalline samples of **1′** and **3′** were grown at room temperature over 1-2 days while samples of **2′** were grown at −35°C over 1-2 days. These crystalline samples were ground into fine microcrystalline powder and loaded into 4 mm OD quartz tubes (Wilmad 707-SQ-250M). The solid powder was then restrained with eicosane and flame-sealed under vacuum (<60 mTorr).

Continuous wave EPR measurements.

The cw-EPR measurements for **1′**-**3′** were performed at the Massachusetts Institute of Technology with a Bruker EMX-Plus spectrometer with an ER4119HS high sensitivity X-band resonator for perpendicular mode. All measurements were performed at 4-5 K using a Bruker/ColdEdge 4K waveguide cryogen-free cryostat.

Pulsed EPR measurements.

Pulsed EPR measurements for **1′**-**3′** were performed at the Massachusetts Institute of Technology on a Bruker ElexSys E580 X-band EPR spectrometer using an EN 4118X-MD4 ENDOR probe. Temperature was controlled using a Stinger ColdEdge 4K Flow System. All EPR data were processed by a combination of Python, Matlab,[7] Easyspin,[8] and Origin.[9] Absolute intensities of the cw-EPR spectra were normalized between 0 and 1 prior to simulation of the data in EasySpin using the pepper function. All cw-EPR data were simulated the resulting spectra with the spin Hamiltonian $\hat{H} = D(\hat{S}_z^2 - S(S+1)/3) + E(\hat{S}_x^2 - \hat{S}_y^2) + \sum_i g_{iso}\mu_B \hat{S}_i B_i$, where D and E are the axial and transverse zero-field splitting (ZFS) parameters, respectively, μ_B is the Bohr magneton,

S_i is the spin operator, g_{iso} is the isotropic electron g-value, and B_0 the magnetic field. (see Table S6 for full fitting parameters).

X-ray Structure Determination.

Single crystal X-ray diffraction data for **1**, **3**, and **1-Al** were collected using a STOE STADIVARI diffractometer using Axo microfocus CuKa source, an EIGER2 1M CDTE detector, and an Oxford Cryostream cooler. Raw data were integrated using X-Area. Absorption corrections were applied by Gaussian integration in STOE X-Red32. Scaling of reflection intensities was performed with STOE LANA. All samples were coated in Paratone N oil and mounted on a MiTeGen MicroLoop™. Single crystal X-ray diffraction data for **2** (previously reported structure at room temperature K)[2] and **3-Al** were collected in the X-ray crystallography lab of the Integrated Molecular Structure Education and Research Center (IMSERC) at Northwestern University. All samples were coated in Paratone N oil and mounted on a MiTeGen MicroLoop™. Crystallographic data for **2** were collected on a Bruker KAPPA diffractometer with a MoKa IμS microfocus X-ray source with Quazar Optics, Apex II detector, and an Oxford Cryosystems Cryostream cryostat. Raw data were integrated using SAINT V8.30 and Absorption corrections were applied using SADABS V2.03. Crystallographic data for **3-Al** were collected on a Rigaku XtaLAB Synergy (Single source) with a micro-focus sealed X-ray tube PhotonJet (MoKa) radiation source, HyPix CCD detector and an Oxford Cryostream cooler. Raw data were integrated using CrysAlisPro. Absorption corrections were applied using multi-scan absorption correction with the SCALE3 ABSPACK module in CrysAlisPro.[10]

The space groups of each compound were determined by examination of systematic absences, E-statistics, and successive refinement of the structure. Using the OLEX2 interface,[11] the structures were solved with intrinsic phasing (SHELXT) methods[12] and further refined using least squares minimization with SHELXL.[13] Thermal parameters for all non-hydrogen atoms were refined anisotropically. All hydrogen atoms were fixed at ideal positions, refined using a riding model for all structures, and refined using isotropic displacement parameters derived from their parent atoms. Crystallographic details are listed in Tables S1–S5.

Steady-State Photoluminescence Measurements.

Low-temperature photoluminescence measurements were performed in a closed-cycle cryostat (Montana Instruments, Cryostation s100) with free space optical access (**1′-3′**), a helium-flow cryostat (Janis ST-500) with free space optical access (**4′**), or in a helium bath (**5′, 6′**) with optical

fiber access. **1′-3′** were excited at 785 nm (Thorlabs, FPL785S-250) through an objective (Olympus, LCPLN100XIR) mounted inside the cryostat. **4′-6′** were excited at 660 nm (Thorlabs S1FC660) either through an optical fiber (Thorlabs, FP400ERT) mounted directly on the sample (**4′**) or a lens external to the cryostat (**5′, 6′**). Shortpass filters were used to clean-up the excitation beams. A broadband 50:50 beamsplitter (BS, Thorlabs, BSW29R) was used to separate excitation and collection paths, and laser scatter was filtered using longpass filters. The PL was coupled into a multi-mode optical fiber and sent to a spectrometer (Acton, SpectraPro 2500i) equipped with an InGaAs detector (Princeton Instruments, Pylon-IR or OMA V:1024-2.2) to record spectra.

Other Physical Measurements.

Nuclear magnetic resonance experiments were conducted on a two-channel Bruker Avance-III HD Nanobay spectrometer operating at 400 MHz. For ^{27}Al NMR experiments, samples were dissolved in THF-h_8 to ensure sufficient signal from the ionic Al compounds. Ultraviolet-visible-near infrared electronic absorption (UV-Vis-NIR) spectra were collected on 0.1-0.6 mM samples of **1-3** in THF with a Varian Cary 5000 spectrophotometer. Molar absorptivity values were determined from Beer-Lambert law in the 0.05-0.6 mM range, where the raw absorbance values increased linearly with concentration and were <1. Inductively coupled plasma optical emission spectrometry (ICP-OES) measurements were collected with a Thermo iCAP 7600 instrument to determine molar ratios for EPR samples.

Additional computational details.

Spin-polarized DFT calculations were performed using VASP 6.3.2[14–17] with projector augmented wave pseudopotentials[18,19] and the PBE exchange correlation functional[20,21]. For **k**-point integrations, Gaussian smearing of 0.05 eV was used. We used a 600 eV energy cutoff for the planewave expansion and an energy convergence of 1×10^{-7} eV. For neutral molecules, **2**, calculations were performed on a single molecule with 14 Å of vacuum between periodic images. For charged molecules, **1** and **3**, calculations were performed on a unit cell including both the molecular color center and its counterion. For Figure 4, orbitals are only visualized if they contain at least 50% molecular color center character.

Excited state calculations were performed using the ΔSCF method.[22,23] We calculate the excited state singlet-triplet gap by subtracting the total energy of a constrained occupancy calculation, where the electron in the spin-up HOMO is promoted to the spin-down LUMO, from a standard ground state DFT calculation. For all calculations, including the constrained occupancy calculations, the relaxed ground state geometry is used.

Supplementary Text

Molecular design rules for optical addressability with resonant excitation: First, we must be able to *selectively* excite from individual ground state spin sublevels into an emissive excited state to initialize the ground state. For $S = 1$ transition metal systems, these emissive transitions generally occur from the spin-singlet excited state (^{1}ES) to the spin-triplet ground state (^{3}ES). By selectively exciting from a particular M_S sublevel into ^{1}ES, coupled with non-selective decay to all ground state spin sublevel, we can optically polarize the spin population into the remaining spin sublevels. The ground state spin lifetime must be longer than the optical lifetime to ensure that the optically driven spin polarization is retained once the excited state population has decayed to the ground state. We must then coherently control this ground state with microwaves, followed by optical readout. The optical photons emitted from the ^{1}ES during this process act as a handle to read out the evolution of the spin state both during the initialization process (*e.g.,* hole burning) and following coherent spin manipulation (*e.g.,* Hahn-echo experiments). To allow for emission from this ^{1}ES, we need to eliminate lower energy spin-triplet excited states (^{3}ES) that provide non-radiative decay pathways so the ^{1}ES is the lowest energy excited state.[24] This feature requires a sufficiently strong ligand field around the spin-bearing metal center.[25] The resulting optical-spin interface offers a potential route to reach single spin detection and readout.

In contrast, optical addressability may be achieved through off-resonant excitation if the system contains a spin-selective optical relaxation process. The prototypical example among quantum sensors is the anionic nitrogen vacancy center in diamond.[26,27] In this case, excitation from a ground state triplet into the excited state triplet manifold is followed by spin-selective intersystem crossing to the singlet excited state manifold. This mechanism creates two (or more) different relaxation rates from the excited state triplet to the ground state triplet manifold. Additionally, one relaxation pathway is spin conserving (^{3}ES$\rightarrow$^{3}GS) while the other is spin non-conserving (^{3}ES$\rightarrow$^{1}ES$\rightarrow$^{3}ES) such that certain spin sublevels will retain their spin population over the course of the optical cycling while the spin population that goes through the intersystem crossing mechanism may be shuffled into other spin sublevels. These systems therefore have similar requirements to resonant excitation schemes, such as a ground state lifetime that is much shorter than the ground

state spin lifetime, but distinctly, do not require resonant laser excitation or narrow optical linewidths.

Power-dependent EPR measurements: As mentioned in the main text, power-dependent pulsed EPR measurements provide a mechanism to mitigate the influence of direct coupling between nearby paramagnetic centers, referred to as instantaneous diffusion, on spin coherence.[28–30] The reduction in microwave power should result in increasingly small tipping angles from the microwave pulses in the Hahn-echo sequence. The extrapolation of these measurements to infinitely small tipping angles should, in principle, provide coherence times that are independent of instantaneous diffusion. This approach should be regarded as an estimate, as opposed to an absolute value, of the coherence times in the nuclear spin-limited regime.

The analysis for this measurement occurs as follows. We first perform nutation experiments at variable microwave powers at 6 K to determine the Rabi frequency of the electronic spins in the sample at each microwave power (Figure S2). From these experiments, we determine the tipping angle, θ, at 16 ns. We then perform two pulse Hahn echo experiments (p_1-τ-p_2-τ-echo) at 6 K at the microwave powers as the nutation experiments while fixing the pulse lengths, p_1 and p_2, at 16 and 32 ns, respectively. Using the tipping angle calculated from the nutation experiments, we fit the resulting relaxation times as follows:

$$(1/T_{\mathrm{m,exp}}) = \mathrm{m}(\sin^2(\theta/2)) + (1/T_{\mathrm{m},\theta\to0})$$

where $T_{\mathrm{m,exp}}$ is the measured T_{m} time at each microwave power, θ is the calculated tipping angle from the nutation experiments, m is the slope, and $T_{\mathrm{m},\theta\to0}$ is the y-intercept corresponding to the extrapolated T_{m} time at infinitely θ. The resulting $T_{\mathrm{m},\theta\to0}$ times for **1′**, **2′**, and **3′** are 0.75, 1.92, and 1.03 μs (Figure S2). The T_{m} times are largely invariant to microwave power for **1′** and **3′**, suggesting that the high nuclear spin density, as opposed to the high electronic spin concentration, is limiting coherence. In contrast, **2′** shows a slightly longer $T_{\mathrm{m},\theta\to0}$ of around 2 μs, which falls in a similar range to the coherence times measured for Cr(*o*-tolyl)$_4$. Given that both **2′** and Cr(*o*-tolyl)$_4$ were measured in the same matrix, Sn(*o*-tolyl)$_4$, and therefore, the same nuclear spin environment, we would expect the Mo^{4+} and Cr^{4+} to exhibit similar T_{m} times in the nuclear-spin limited regime. Thus, **2′** may exhibit slightly longer T_{m} times in more dilute samples (assuming no aggregation of paramagnetic centers during solid-state dilution).

Table S1 | Crystallographic information for the structural refinement of **1**

Empirical Formula	$C_{44}H_{60}VLiO_4$
Formula weight	710.82 g/mol
Temperature	100 K
Wavelength	1.54186 Å
Crystal System	Triclinic
Space Group	P−1
Unit Cell Dimensions	a = 11.487(4) Å, α = 70.281(19) °
	b = 16.971(4) Å, β = 86.57(2) °
	c = 22.100(6) Å, γ = 84.97(2) °
Volume	4038(2) Å^3
Z	4
Density (calculated)	1.168 g/cm^3
Absorption coefficient	2.349 mm^{-1}
F_{000}	1526.0
Crystal color	Violet
θ range	2.773 to 70.665 °
Index ranges	$-9 \leq h \leq 14$
	$-19 \leq k \leq 20$
	$-26 \leq l \leq 26$
Reflections collected	14814
Independent reflections	10141 [R_{int} = 0.0467]
Completeness to θ = 67.686 °	97.6%
Absorption correction	Integration
Refinement method	Full-matrix least-squares on F^2
Data / restraints / parameters	14814 / 0 / 918
Goodness-of-fit on F^{2a}	1.019
Final R indices [I > 4σ(I) = 18299 data]^b	R_1 = 5.91 %, wR_2 = 8.99 %
R indices (all data, 0.80 Å)	R_1 = 16.15 %, wR_2 = 17.99 %
Largest diff. peak and hole	0.757 and –0.442 e/Å^{-3}

^{a} GooF = $[\Sigma[w(F_o^2–F_c^2)^2] / (n-p)]^{1/2}$ where n is the number of reflections and p is the total number of parameters refined. $^b R_1 = \Sigma||F_o|–|F_c|| / \Sigma|F_o|$; $wR_2 = [\Sigma[w(F_o^2–F_c^2)^2] / \Sigma[w(F_o^2)^2]]^{1/2}$

Table S2 | Crystallographic information for the structural refinement of **2**

Empirical Formula	$C_{28}H_{28}Mo$
Formula weight	460.44 g/mol
Temperature	100 K
Wavelength	0.71073 Å
Crystal System	Tetragonal
Space Group	$P{-}42_1c$
Unit Cell Dimensions	a = 11.8490(2) Å, α = 90°
	b = 11.8490(2) Å, β = 90°
	c = 7.8931(3) Å, γ = 90°
Volume	1108.18(6) Å^3
Z	2
Density (calculated)	1.380 g/cm^3
Absorption coefficient	0.603 mm^{-1}
F_{000}	476
Crystal color	Violet
θ range	2.431 to 36.103 °
Index ranges	$-19 \leq h \leq 19$
	$-19 \leq k \leq 19$
	$-12 \leq l \leq 12$
Reflections collected	2577
Independent reflections	2066 [R_{int} = 0.0434]
Completeness to θ = 25.242°	97.2%
Absorption correction	Multi-scan
Refinement method	Full-matrix least-squares on F^2
Data / restraints / parameters	2577 / 0 / 67
Goodness-of-fit on F^{2a}	1.127
Final R indices [I > 4σ(I) = 18299 data]^b	R_1 = 3.65 %, wR_2 = 8.43 %
R indices (all data, 0.80 Å)	R_1 = 5.05 %, wR_2 = 9.17 %
Largest diff. peak and hole	1.102 and –0.406 e/Å^{-3}

^{a} GooF = $[\Sigma[w(F_o^2 - F_c^2)^2] / (n-p)]^{1/2}$ where n is the number of reflections and p is the total number of parameters refined. $^b R_1 = \Sigma||F_o|-|F_c|| / \Sigma|F_o|$; $wR_2 = [\Sigma[w(F_o^2-F_c^2)^2] / \Sigma[w(F_o^2)^2]]^{1/2}$

Table S3 | Crystallographic information for the structural refinement of **3**

Empirical Formula	$C_{32}H_{76}VLiO_8Si_4$
Formula weight	759.17 g/mol
Temperature	249.99(13) K
Wavelength	0.71073 Å
Crystal System	Monoclinic
Space Group	$P2_1/c$
Unit Cell Dimensions	a = 10.4055(2) Å, α = 90°
	b = 21.3852(3) Å, β = 104.167(2)°
	c = 20.4877(4) Å, γ = 90°
Volume	4420.34(14) Å^3
Z	4
Density (calculated)	1.141 g/cm^3
Absorption coefficient	0.373 mm^{-1}
F_{000}	1656.0
Crystal color	Violet
θ range	2.163 to 31.100°
Index ranges	$-14 \leq h \leq 14$
	$-30 \leq k \leq 28$
	$-28 \leq l \leq 26$
Reflections collected	66678
Independent reflections	11931 [R_{int} = 0.041]
Completeness to θ = 26.32°	99.85%
Absorption correction	Multi-scan
Refinement method	Full-matrix least-squares on F^2
Data / restraints / parameters	11931 / 0 / 247
Goodness-of-fit on F^{2a}	1.058
Final R indices [$I > 4\sigma(I)$ = 11931 data]^b	R_1 = 3.22 %, wR_2 = 7.87 %
R indices (all data, 0.80 Å)	R_1 =4.38 %, wR_2 = 8.28 %
Largest diff. peak and hole	0.390 and –0.362 e/Å^{-3}

^{a} GooF = $[\Sigma[w(F_o^2-F_c^2)^2] / (n-p)]^{1/2}$ where n is the number of reflections and p is the total number of parameters refined. $^b R_1 = \Sigma||F_o|-|F_c|| / \Sigma|F_o|$; $wR_2 = [\Sigma[w(F_o^2-F_c^2)^2] / \Sigma[w(F_o^2)^2]]^{1/2}$

Table S4 | Crystallographic information for the structural refinement of **1-Al**

Empirical Formula	$C_{44}H_{60}AlLiO_4$
Formula weight	686.84 g/mol
Temperature	100 K
Wavelength	1.54186 Å
Crystal System	Triclinic
Space Group	P−1
Unit Cell Dimensions	a = 11.461(6) Å, □ = 70.41(4)°
	b = 16.903(11) Å, □ = 86.22(4)°
	c = 22.031(10) Å, □ = 85.05(5)°
Volume	4003(3) $Å^3$
Z	4
Density (calculated)	1.135 g/cm^3
Absorption coefficient	0.743 mm^{-1}
F_{000}	1488.0
Crystal color	Colorless
□ range	5.50 to 70.51°
Index ranges	$-13 \leq h \leq 13$
	$-20 \leq k \leq 13$
	$-26 \leq l \leq 20$
Reflections collected	14313
Independent reflections	7669 [R_{int} = 0.0554]
Completeness to θ = 69.796°	94.8%
Absorption correction	Integration
Refinement method	Full-matrix least-squares on F^2
Data / restraints / parameters	14313 / 2 / 918
Goodness-of-fit on F^{2a}	0.795
Final R indices [I > 4σ(I) = 18299 data]^b	R_1 = 4.32 %, wR_2 = 9.49 %
R indices (all data, 0.80 Å)	R_1 = 8.61 %, wR_2 = 10.26 %
Largest diff. peak and hole	0.452 and – 0.208e/$Å^{-3}$

^{a} GooF = $[\Sigma[w(F_o^2 - F_c^2)^2] / (n-p)]^{1/2}$ where n is the number of reflections and p is the total number of parameters refined. $^b R_1 = \Sigma||F_o|-|F_c|| / \Sigma|F_o|$; $wR_2 = [\Sigma[w(F_o^2 - F_c^2)^2] / \Sigma[w(F_o^2)^2]]^{1/2}$

Table S5 | Crystallographic information for the structural refinement of **3-Al**

Empirical Formula	$C_{32}H_{76}AlLiO_8Si_4$
Formula weight	735.20 g/mol
Temperature	100 K
Wavelength	1.54186 Å
Crystal System	Monoclinic
Space Group	$P2_1/c$
Unit Cell Dimensions	a = 10.4242(13) Å, α= 90°
	b = 21.275(2)Å, β = 104.574(10)°
	c = 20.498(3) Å, γ = 90°
Volume	4399.6(9) Å^3
Z	4
Density (calculated)	1.11 g/cm^3
Absorption coefficient	1.770 mm^{-1}
F_{000}	1616
Crystal color	Colorless
θ range	3.046 to 70.009°
Index ranges	$-12 \le h \le 12$
	$-19 \le k \le 25$
	$-19 \le l \le 24$
Reflections collected	8151
Independent reflections	5858 [R_{int} = 0.0345]
Completeness to θ = 67.686°	99.0%
Absorption correction	Integration
Refinement method	Full-matrix least-squares on F^2
Data / restraints / parameters	8151 / 0 / 427
Goodness-of-fit on F^{2a}	0.892
Final R indices [I > 4σ(I) = 18299 data]^b	R_1 = 2.47 %, wR_2 = 5.84 %
R indices (all data, 0.80 Å)	R_1 = 4.63 %, wR_2 = 6.32 %
Largest diff. peak and hole	0.301 and –0.293 e/Å^{-3}

^{a} GooF = $[\Sigma[w(F_o^2-F_c^2)^2] / (n-p)]^{1/2}$ where n is the number of reflections and p is the total number of parameters refined. $^b R_1 = \Sigma||F_o|-|F_c|| / \Sigma|F_o|$; $wR_2 = [\Sigma[w(F_o^2-F_c^2)^2] / \Sigma[w(F_o^2)^2]]^{1/2}$

Table S6 | X-band cw-EPR simulated parameters using EasySpin for **1′-3′**.

Sample	|*D*| (GHz)	|*E*| (GHz)	*g*	*A* (MHz)	Linewidth (lw)
**1′**	5.62	0.79	1.98	[155]	[0 8]
**2′**	7.30	0	1.93	N/A	[0 10]
**3′**	16.6	2.2	[1.91 1.95 1.94]	[165]	[0 5]
**3′ *S*=1/2 impurity**	N/A	N/A	[2.05 2.09]	[0 165]	[0 5]

Table S7 | Structural parameters, structural analyses (refs. 27 and 28) and ZFS values for all compounds

	**∠C_R-M-C_R**						
**Compound**	**Min.**	**Max.**	**τ_4**	**$\tau_{4'}$**	**|*D*| (GHz)**	**|*E*| (GHz)**	***E*/*D***
**1**	104.82	112.38	1.01	1.00	5.62	0.79	0.14
**2**	107.43	113.64	0.98	0.96	7.30	0	0
**3**	105.14	115.77	0.98	0.95	16.6	2.2	0.13
**4**	102.4	113.1	1.022	0.988	3.53[(a)]	0 [(a)]	0
**5**	107.3	113.5	0.984	0.964	1.23[(a)]	0.11[(a)]	0.09

[(a)] ZFS values from ref. 23

Table S8 | Spin active compound:diamagnetic host ratios determined from ICP-OES and calculated molarities.

Sample	Qubit:Host (w/w %)
**1′**	1.5%
**2′**	0.36%
**3′**	3.0%

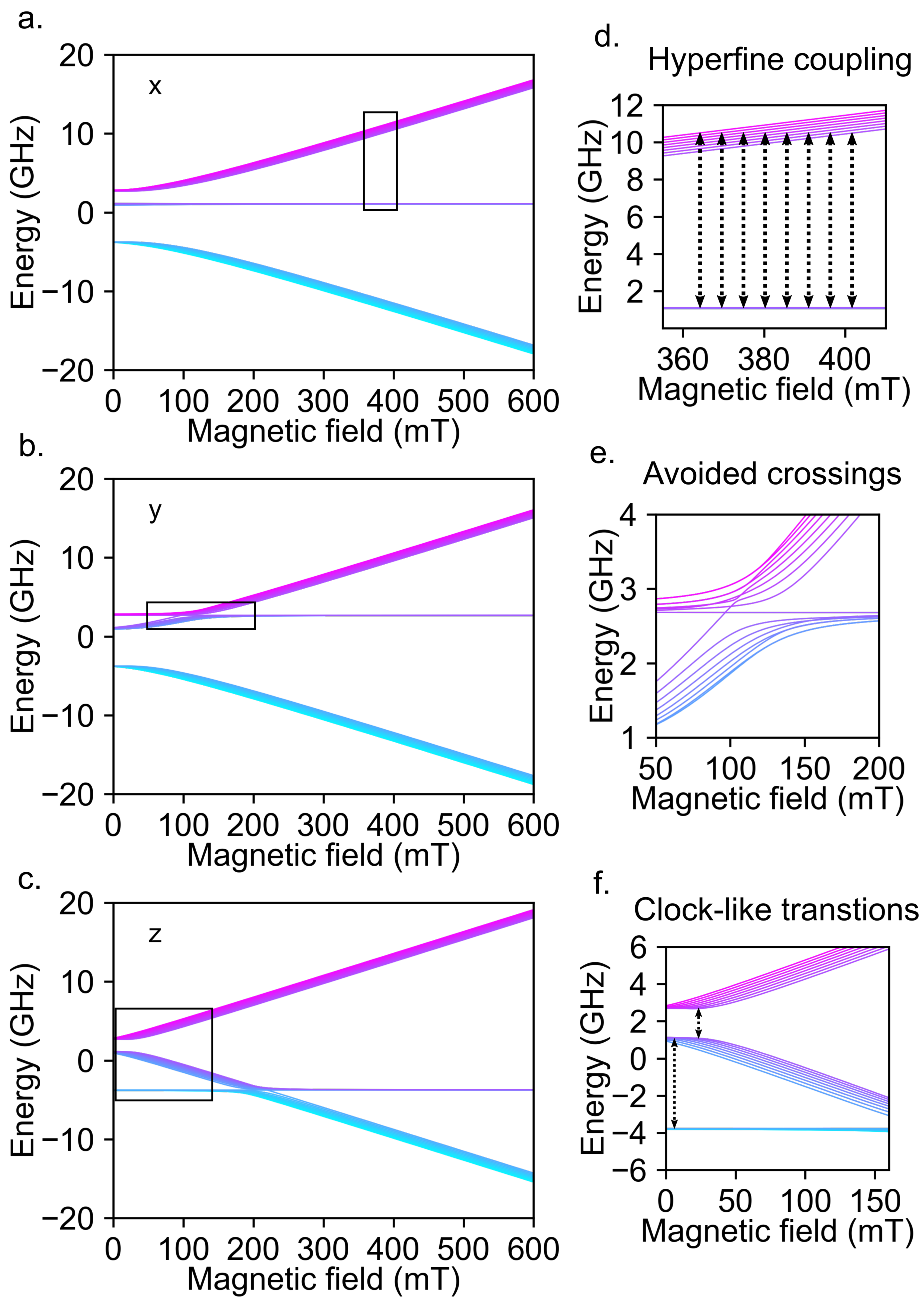

Figure S1 | Zeeman splitting diagrams for [Li(THF)$_4$][V(*o*-tolyl)$_4$] in [Li(THF)$_4$][Al(*o*-tolyl)$_4$] (sample **1′** in the main text) depicting the field-dependent variation of the spin sublevels along x (a), y (b), and z (c). (d) Zoomed in depiction of the boxed area in (a), highlighting the result of hyperfine coupling to the I = 7/2 nucleus. (e) Zoomed in depiction of the boxed area in (b), highlighting the avoided crossings around 100 mT. (f) Zoomed in depiction of the boxed area in (c), highlighting the clock-like transitions around 0 mT.

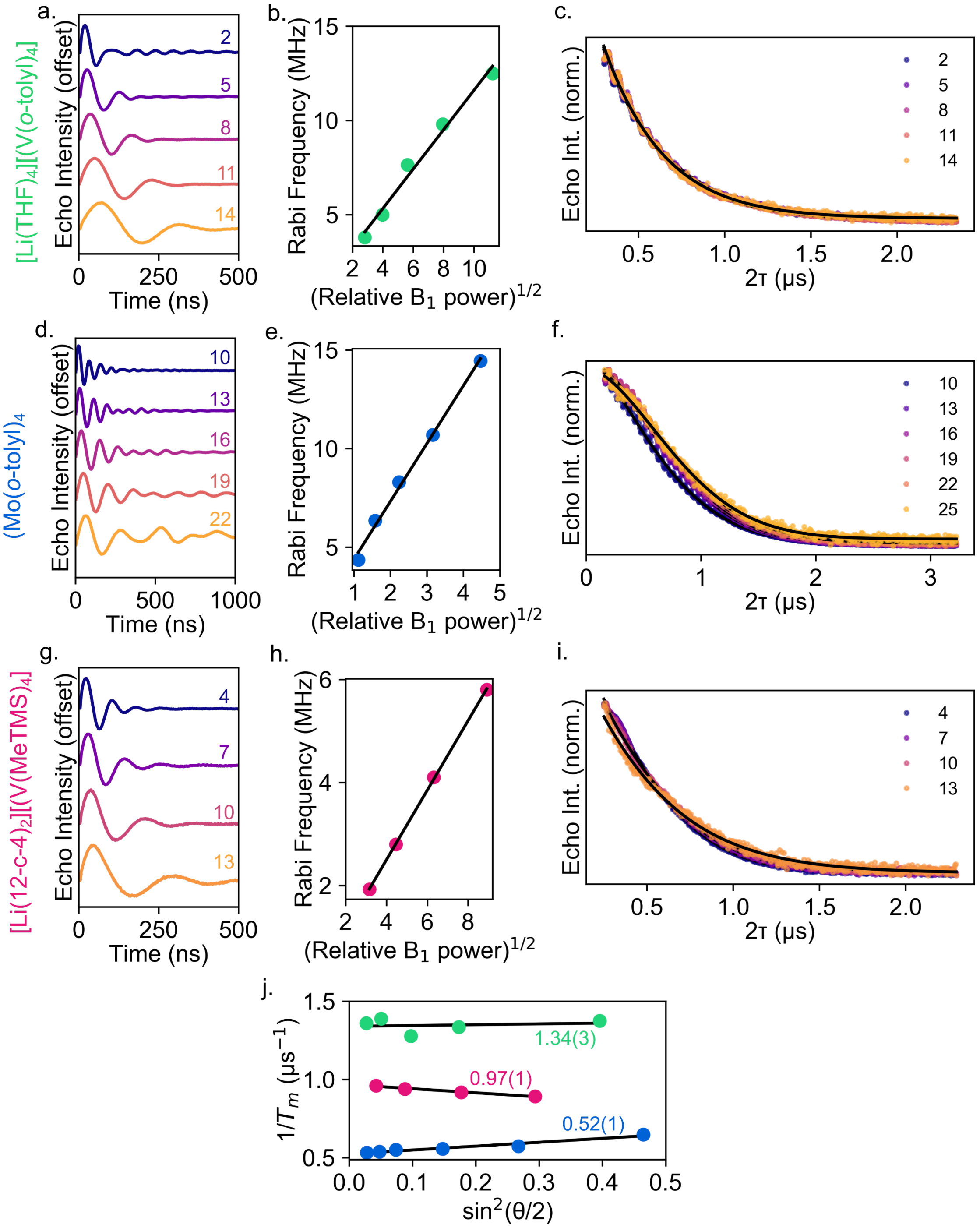

Figure S2 | 6 K nutation experiments at 9.7 GHz for **1′** at 420 mT (a), **2′** at 460 mT (d), and **3′** at 930 mT (g) with microwave attenuations shown in dB. Fast Fourier Transforms of these experiments yielded the Rabi frequency for **1′** (b), **2′** (e), and **3′** (h) to show the expected linear dependence of Rabi frequency on the square root of microwave power. Hahn echo decay curves measured at each microwave power **1′** (c), **2′** (f), and **3′** (j) used to determine T_m times. (j) Linear fits of $1/T_m$ as a function of the tipping angle, θ, for **1′** (green), **2′** (blue), and **3′** (pink) with the y-intercept shown. Error for the y-intercept given in parentheses.

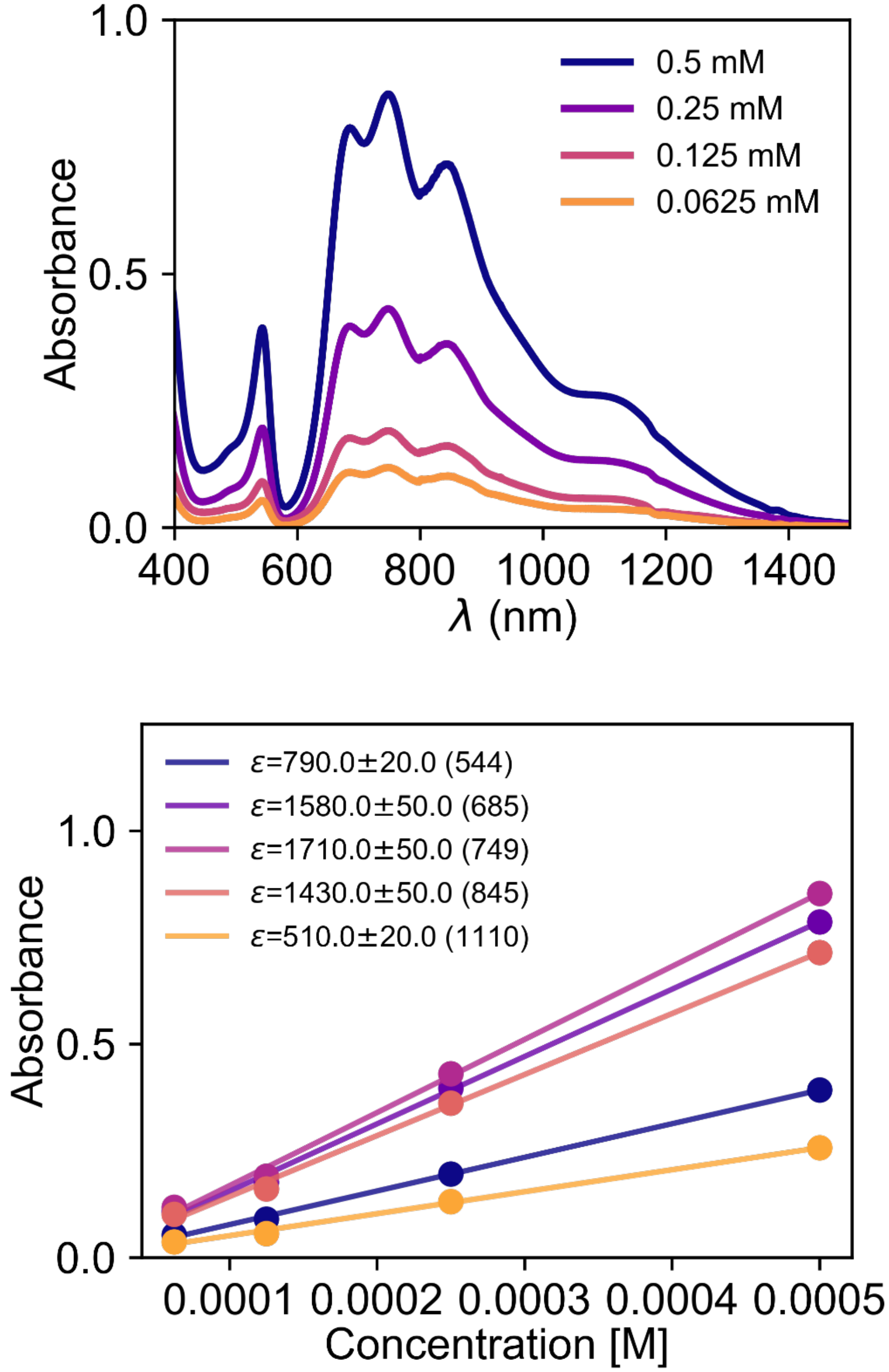

Figure S3 | (top) Electronic absorption spectra of $[Li(THF)_4][V(o\text{-tolyl})_4]$ (**1**) in THF at room temperature. Concentrations indicated in the legend. (bottom) Plot of absorbance maxima versus concentration used to extract molar absorptivity values, ε (M^{-1} cm^{-1}), given in the legend with the value in parentheses corresponding to the wavelength in nm.

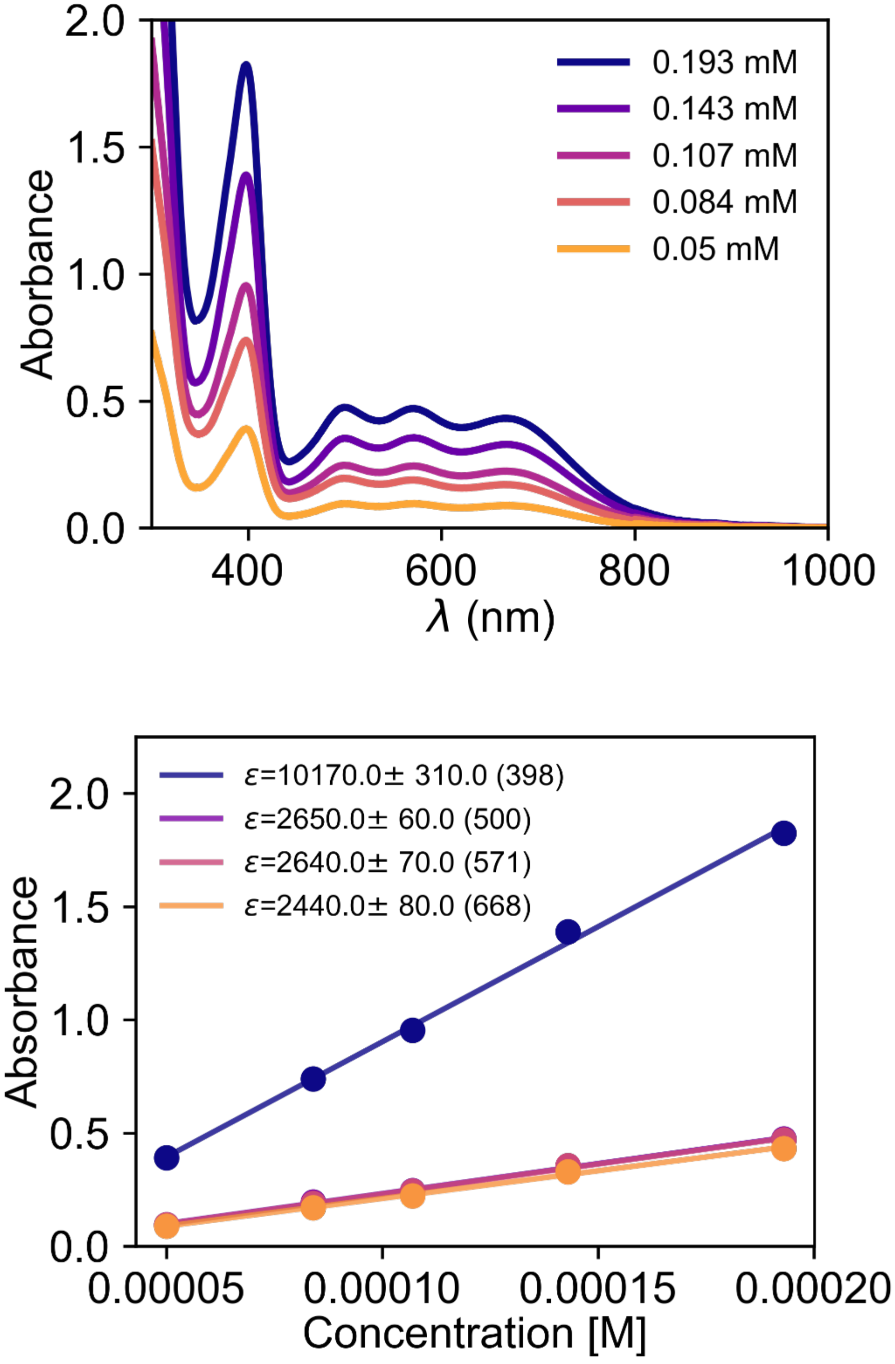

Figure S4 | (top) Electronic absorption spectra of Mo(*o*-tolyl)$_4$ (**2**) in THF at room temperature. Concentrations indicated in the legend. (bottom) Plot of absorbance maxima versus concentration used to extract molar absorptivity values, ε (M^{-1} cm^{-1}), given in the legend with the value in parentheses corresponding to the wavelength in nm.

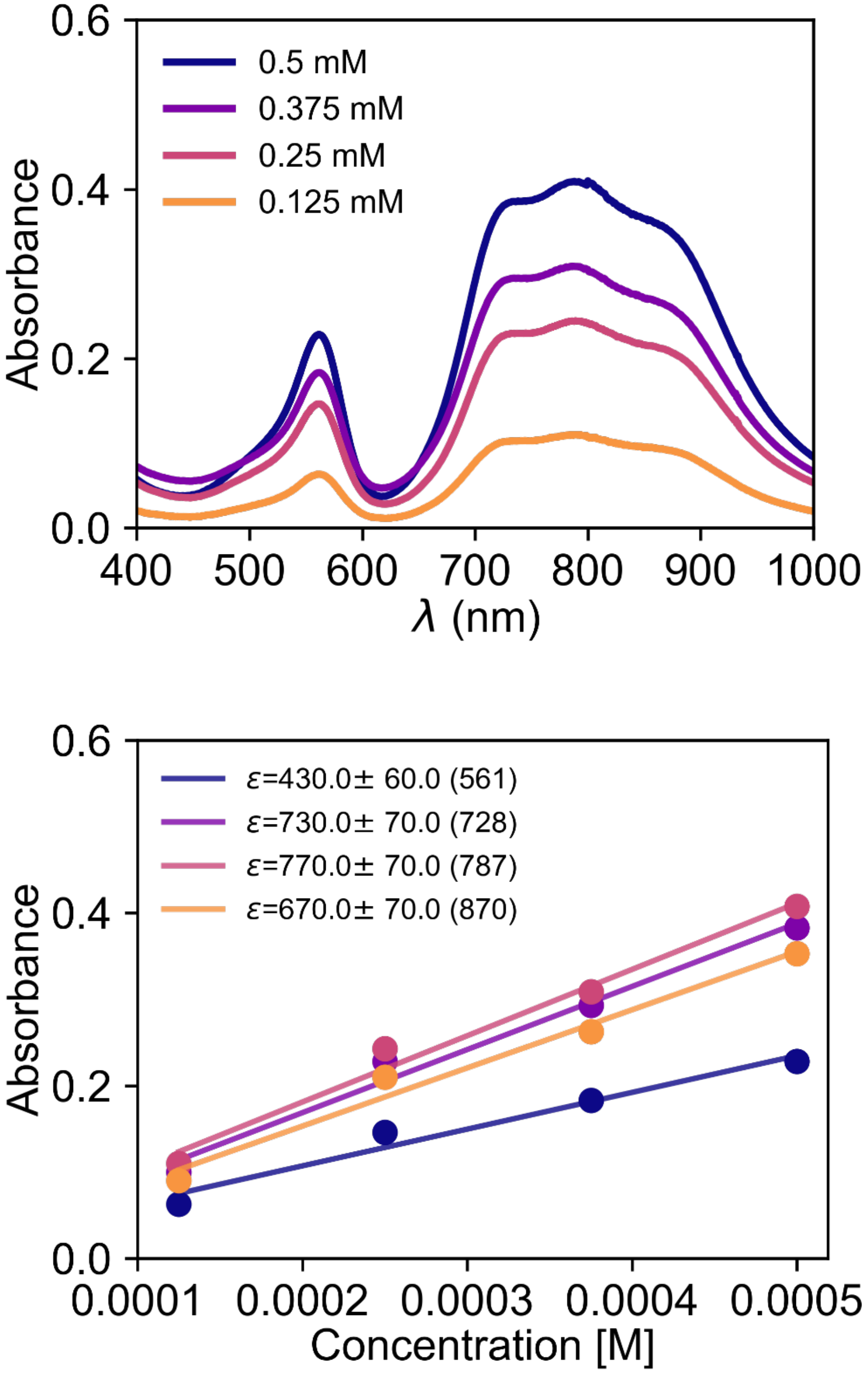

Figure S5 | (top) Electronic absorption spectra of [Li(12-crown-4)$_2$][V(methyltrimethylsilyl)$_4$] (**3**) in THF at room temperature. Concentrations indicated in the legend. (bottom) Plot of absorbance maxima versus concentration used to extract molar absorptivity values, ε (M^{-1} cm^{-1}), given in the legend with the value in parentheses corresponding to the wavelength in nm.

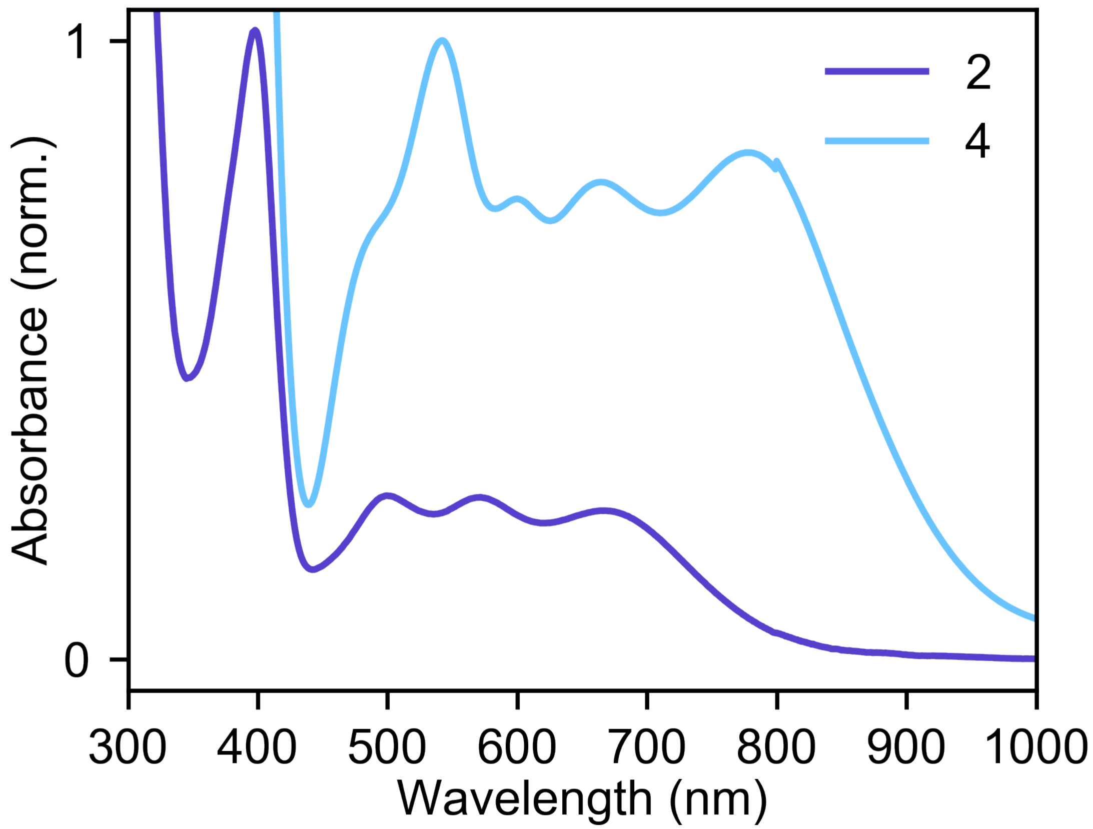

Figure S6 | Comparison of UV-Vis-NIR spectra of Mo(*o*-tolyl)$_4$ (**2**) and Cr(*o*-tolyl)$_4$ (**4**) in THF at room temperature. The spectra are normalized to their most intense features arising from d-d transitions. In each case, the spectra show three lower energy features with a more intense transition around 400 and 525 nm for **2** and **4**, respectively. The intense absorption at wavelengths <350 and <450 nm for **2** and **4**, respectively, likely arises from charge transfer or ligand-centered transitions.

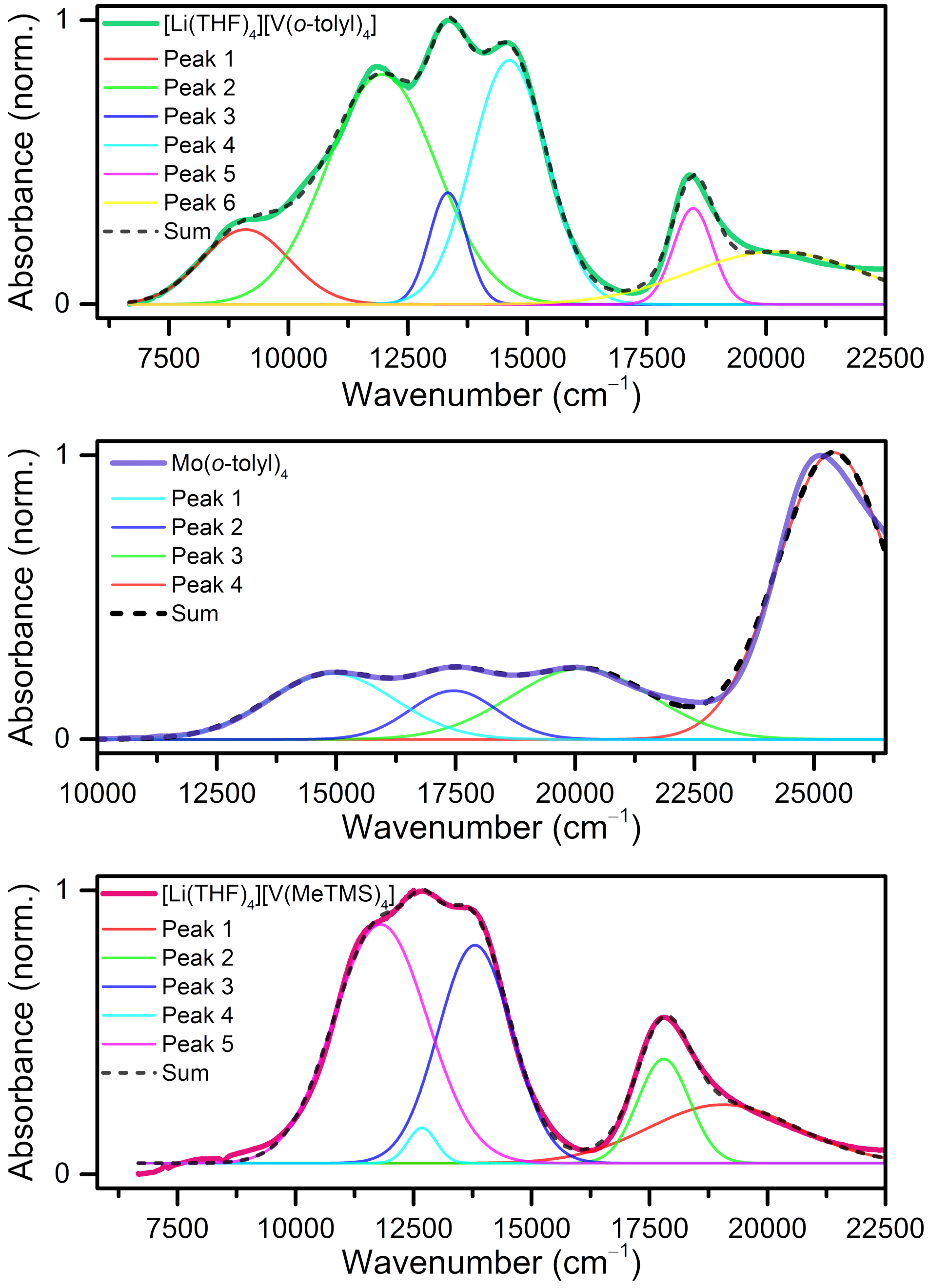

Figure S7 | Peak deconvolution for UV-Vis-NIR absorption spectra of **1-3** in THF at room temperature. Cumulative peak fitting is given as black, dashed line. For the above peak deconvolution, peak maxima (cm^{-1}) with the full-width half maxima value (cm^{-1}) given in parentheses: (top) **1**: 9103 (2138), 11959 (2698), 13339 (918), 14623 (1844), 18471 (965), 20215 (4255); (middle) **2**: 14908 (3094), 17458 (2141), 20136 (3472), 25406 (2760); (bottom) **3**: 11801(2088), 12678 (94), 13801 (1500), 17802 (503), 19055 (811)

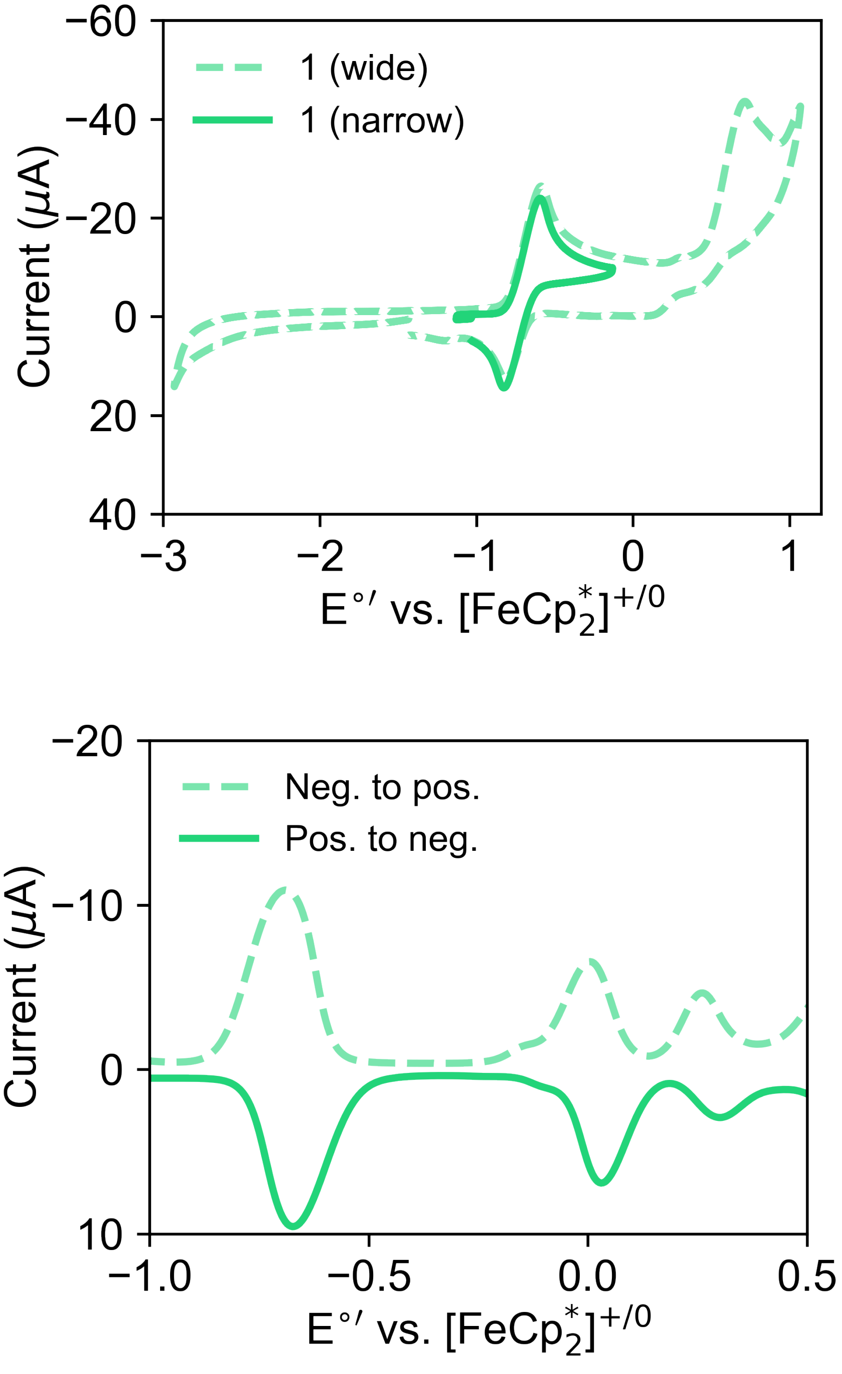

Figure S8 | (top) Cyclic voltammograms of $[Li(THF)_4][V(o\text{-tolyl})_4]$. Scan rate for both experiments was 200 mV/s. (bottom) Differential pulsed voltammograms of $[Li(THF)_4][V(o\text{-tolyl})_4]$, scanning both from −1 V to 0.5 V (Neg. to pos.) and 0.5 V to −1 V (Pos. to neg.). All experiments were performed in THF containing 0.1 M of $[Bu_4N][PF_6]$ and referenced to $[Cp^*{}_2Fe]^{+/0}$. Data were collected using a glassy carbon working electrode, platinum wire counter electrode, and a silver wire pseudo-reference electrode.

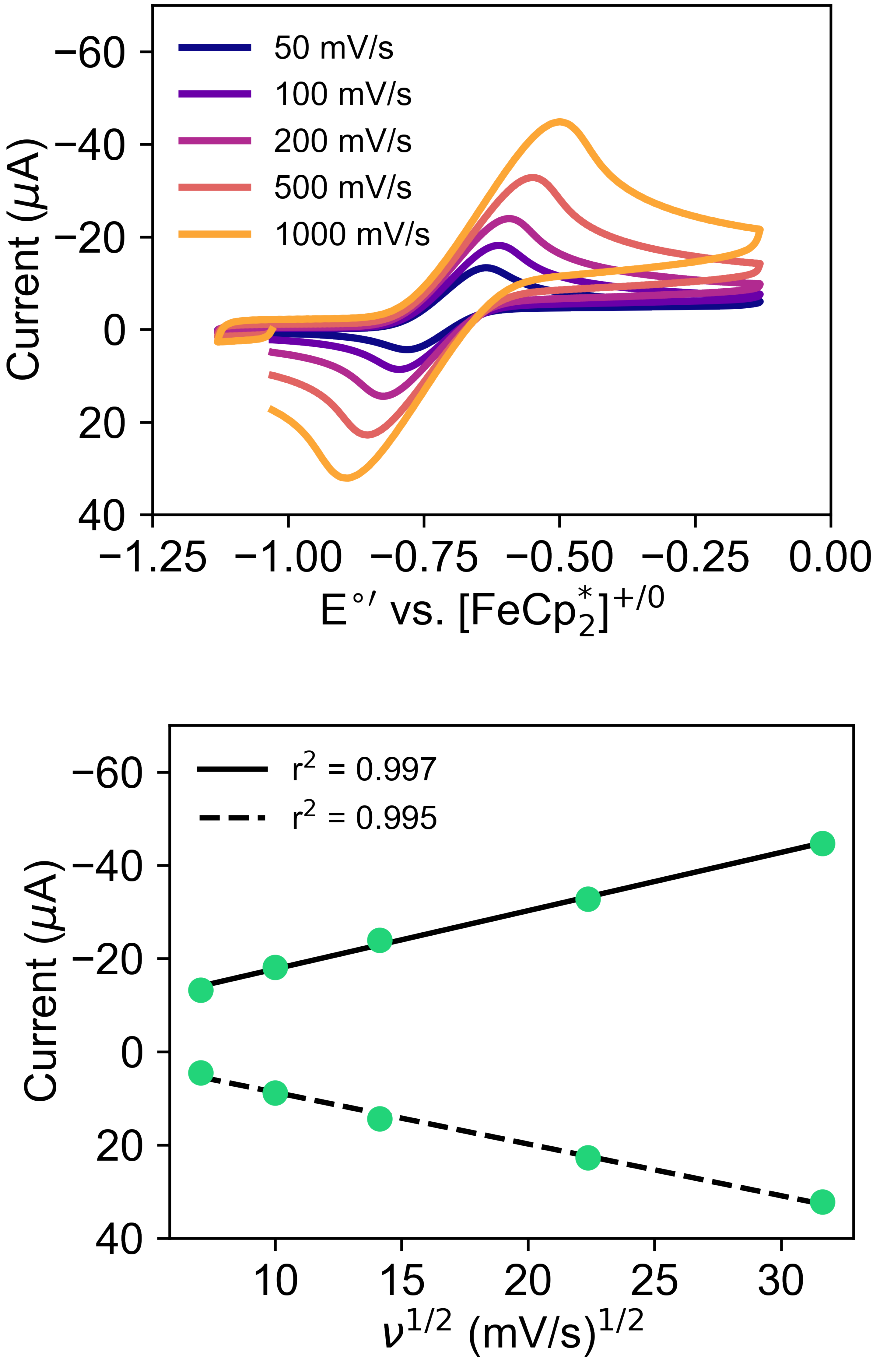

Figure S9 | (top) Scan rate dependence of the $V^{3+/4+}$ redox couple of [Li(THF)$_4$][V(*o*-tolyl)$_4$]. (bottom) Plot of current versus the square root of the scan rate for $V^{3+/4+}$ redox couple in the above experiments. All experiments were performed in THF containing 0.1 M of [Bu$_4$N][PF$_6$] and referenced to [Cp*$_2$Fe]$^{+/0}$. Data were collected using a glassy carbon working electrode, platinum wire counter electrode, and a silver wire pseudo-reference electrode.

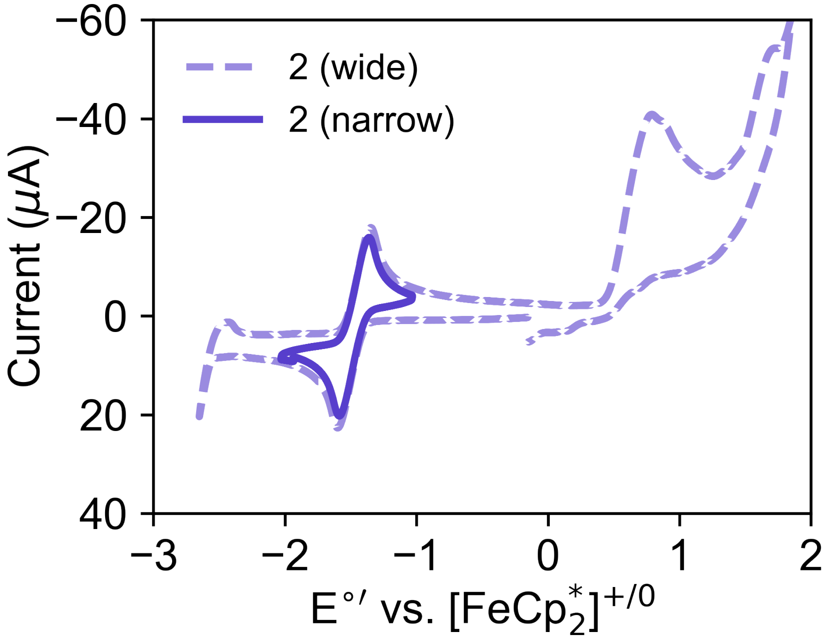

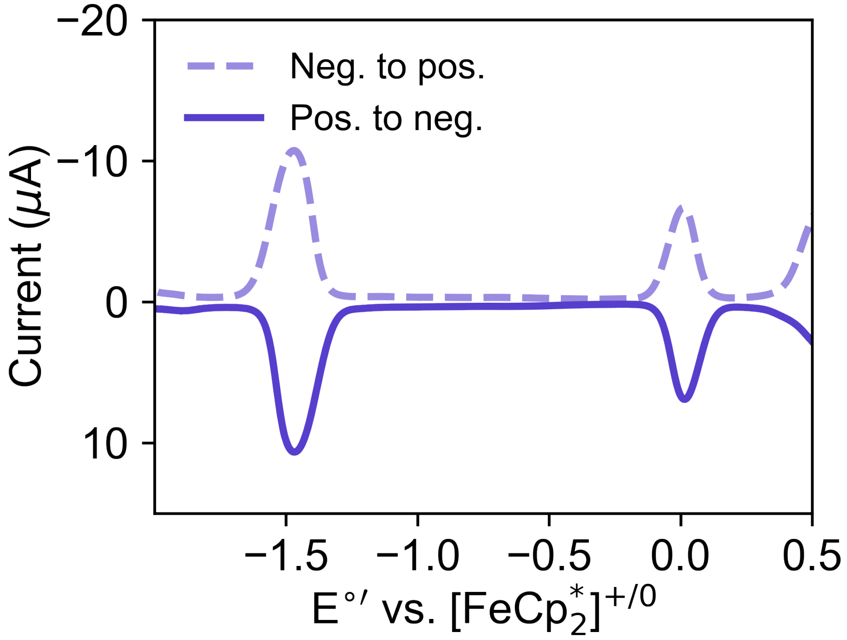

Figure S10 | (top) Cyclic voltammograms of Mo(*o*-tolyl)$_4$. Scan rate for both experiments was 200 mV/s. (bottom) Differential pulsed voltammograms of Mo(*o*-tolyl)$_4$, scanning both from −2 V to 0.5 V (Neg. to pos.) and 0.5 V to −2 V (Pos. to neg.). All experiments were performed in THF containing 0.1 M of $[Bu_4N][PF_6]$ and referenced to $[Cp^*{}_2Fe]^{+/0}$. Data were collected using a glassy carbon working electrode, platinum wire counter electrode, and a silver wire pseudo-reference electrode.

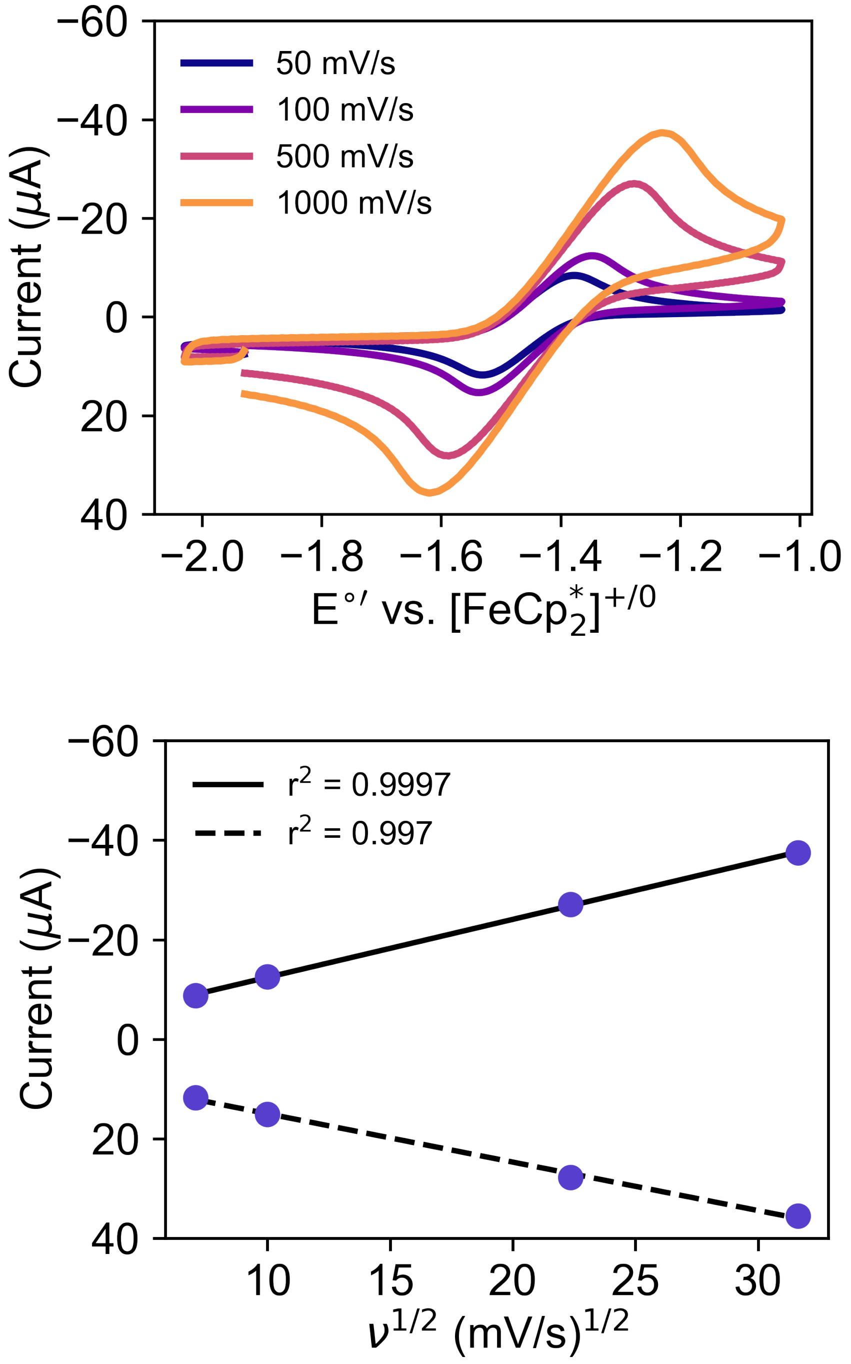

Figure S11 | (top) Scan rate dependence of the $Mo^{3+/4+}$ redox couple of Mo(*o*-tolyl)$_4$. (bottom) Plot of current versus the square root of the scan rate for $V^{3+/4+}$ redox couple in the above experiments. All experiments were performed in THF containing 0.1 M of $[Bu_4N][PF_6]$ and referenced to $[Cp^*_2Fe]^{+/0}$. Data were collected using a glassy carbon working electrode, platinum wire counter electrode, and a silver wire pseudo-reference electrode.

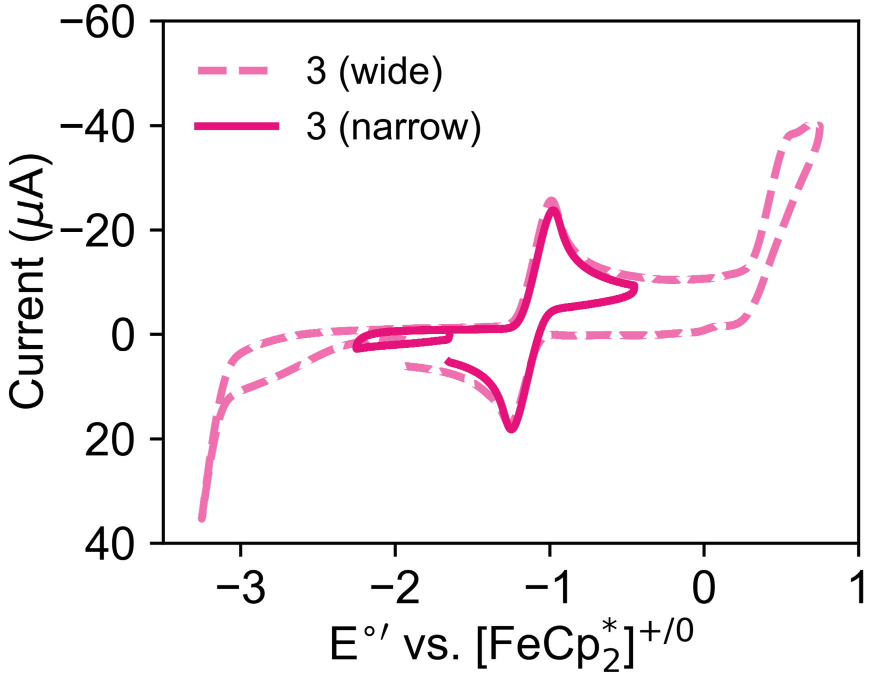

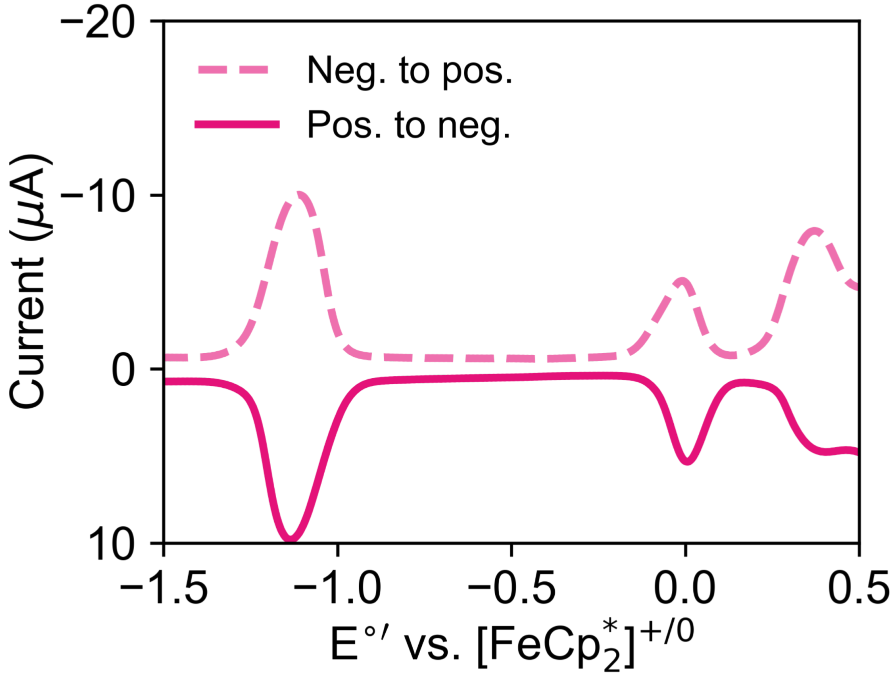

Figure S12 | (top) Cyclic voltammograms of [Li(12-crown-4)$_2$][V(methyltrimethylsilyl)$_4$]. Scan rate for both experiments was 200 mV/s. (bottom) Differential pulsed voltammograms of [Li(12-crown-4)$_2$][V(methyltrimethylsilyl)$_4$], scanning both from −1.5 V to 0.5 V (Neg. to pos.) and 0.5 V to −1.5 V (Pos. to neg.). All experiments were performed in THF containing 0.1 M of [Bu_4N][PF_6] and referenced to [$Cp^*{}_2Fe$]$^{+/0}$. Data were collected using a glassy carbon working electrode, platinum wire counter electrode, and a silver wire pseudo-reference electrode.

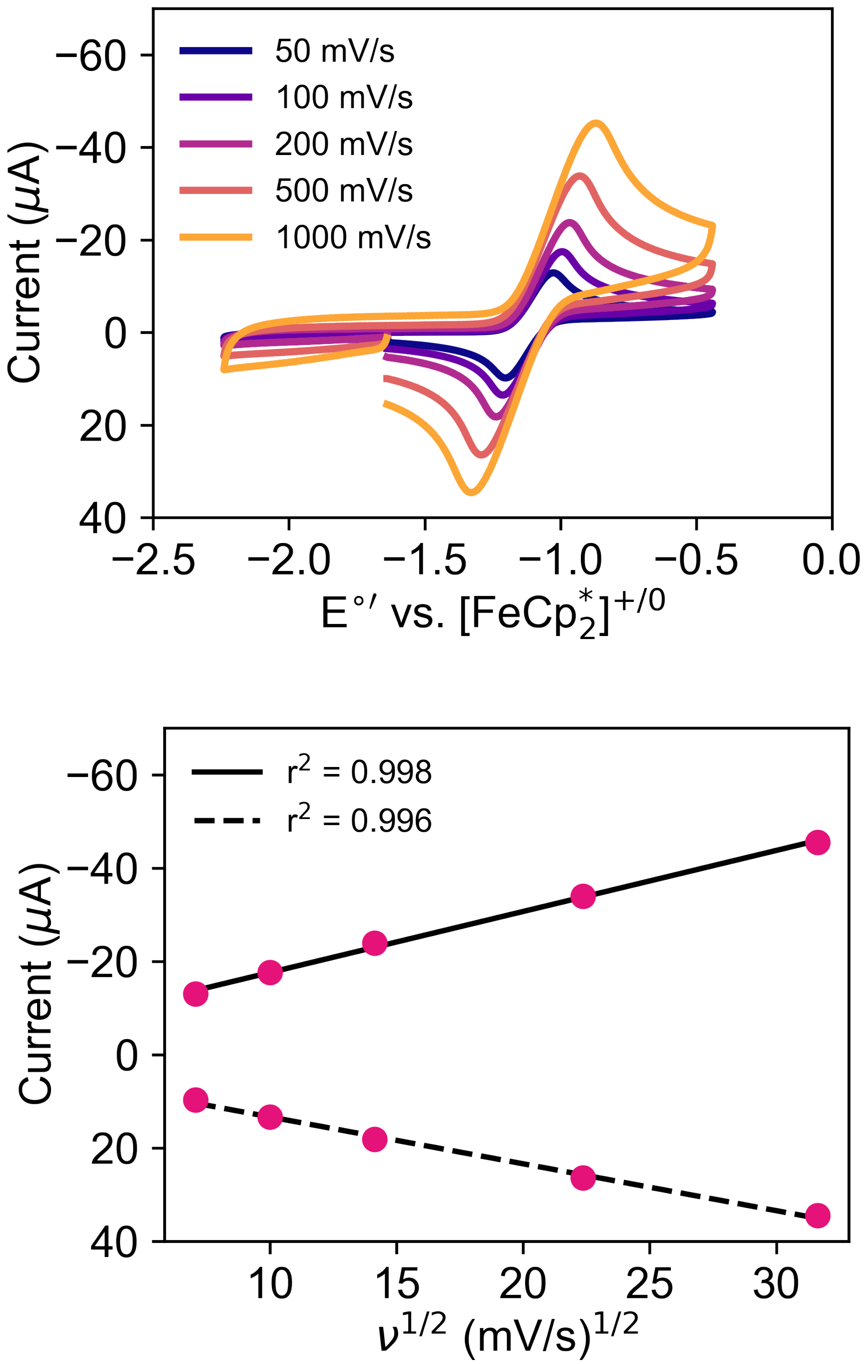

Figure S13 | (top) Scan rate dependence of the $V^{3+/4+}$ redox couple of [Li(12-crown-4)$_2$][V(methyltrimethylsilyl)$_4$]. (bottom) Plot of current versus the square root of the scan rate for $V^{3+/4+}$ redox couple in the above experiments. All experiments were performed in THF containing 0.1 M of [Bu$_4$N][PF$_6$] and referenced to [Cp*$_2$Fe]$^{+/0}$. Data were collected using a glassy carbon working electrode, platinum wire counter electrode, and a silver wire pseudo-reference electrode.

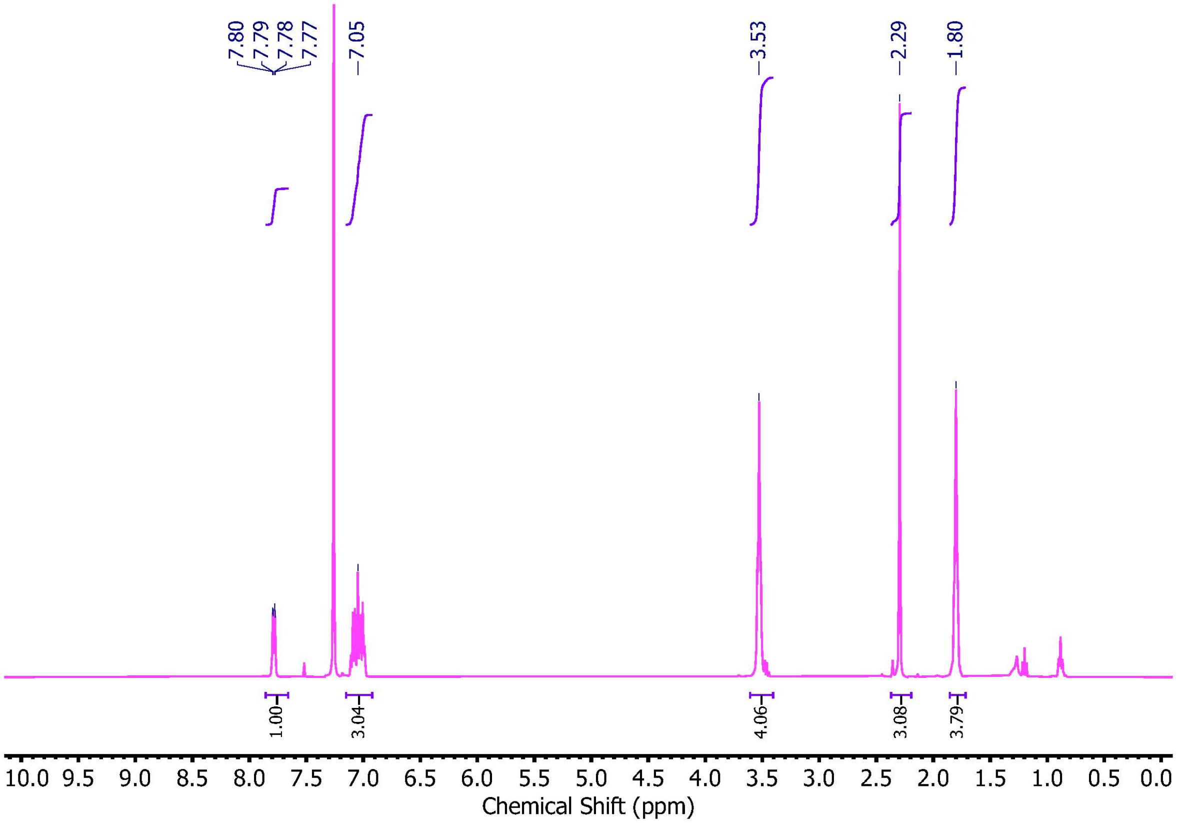

Figure S14 | 400 MHz ^{1}H NMR of **1-Al** in $CDCl_3$. Solvent impurities appear from Et_2O, THF, and hexanes are shown at ~3.5, 1.2-0.7 ppm.

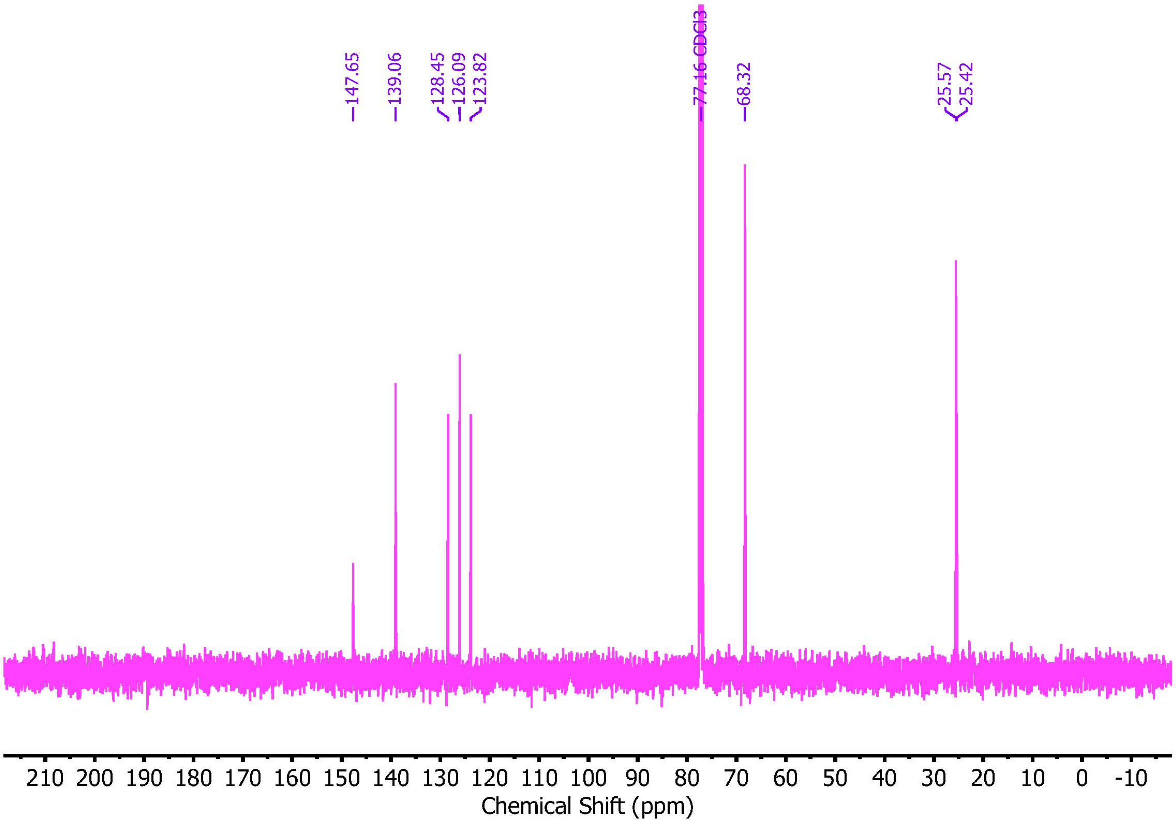

Figure S15 | 101 MHz $^{13}C\{^1H\}$ NMR of **1-Al** in $CDCl_3$.

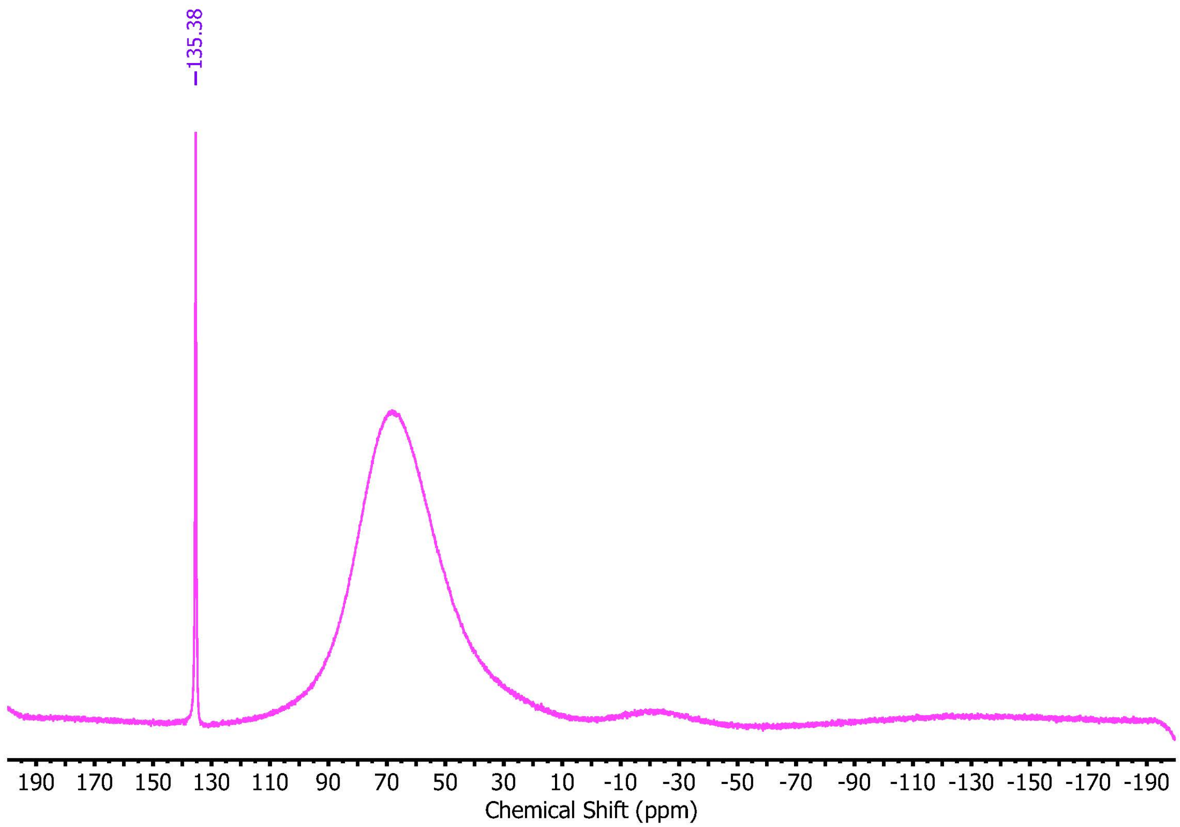

Figure S16 | 104 MHz ^{27}Al NMR of **1-Al** in THF-h_8.

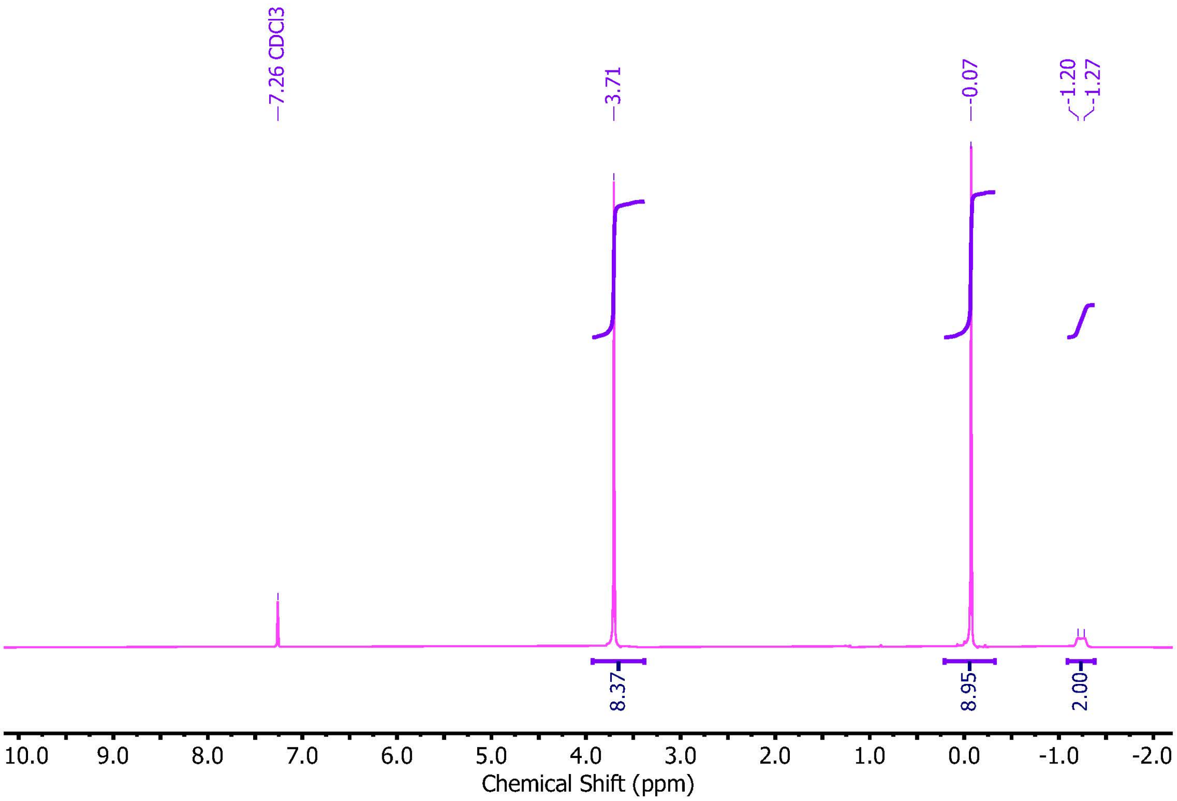

Figure S17 | 400 MHz ^{1}H NMR of **3-Al** in $CDCl_3$.

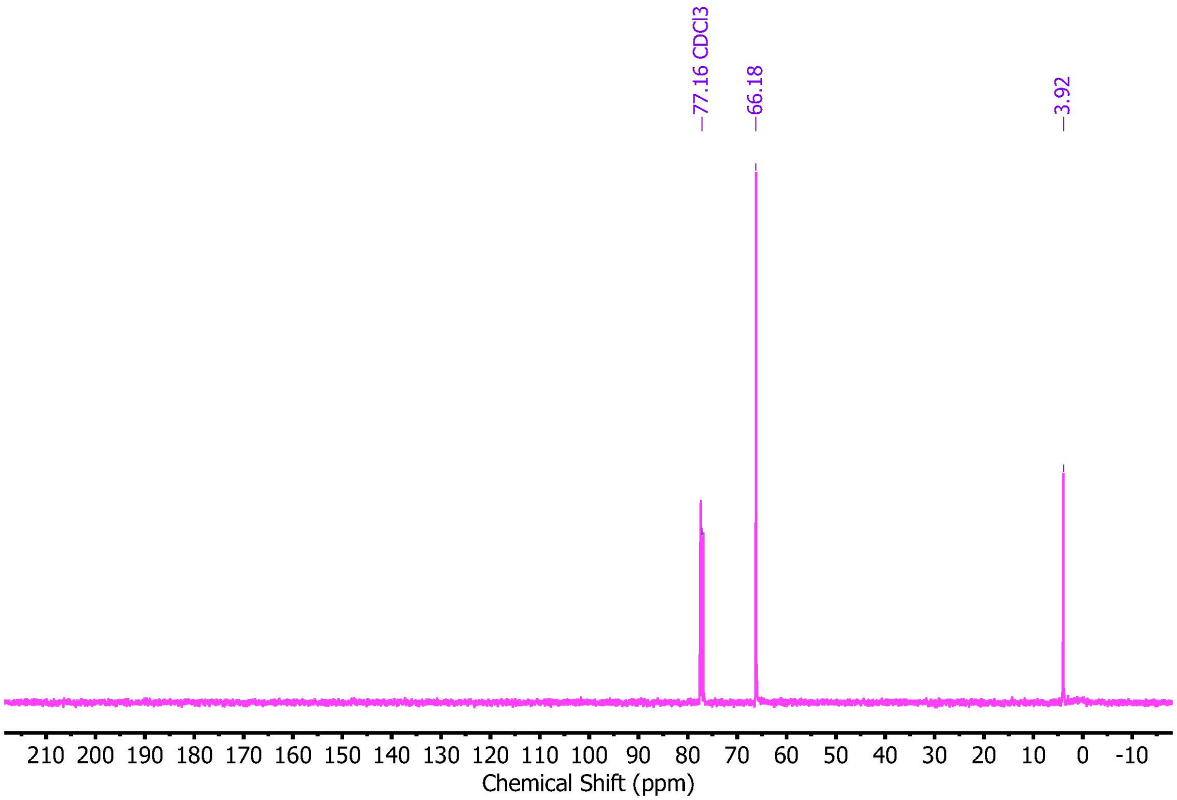

Figure S18 | 101 MHz $^{13}C\{^{1}H\}$ NMR of **3-Al** in $CDCl_3$.

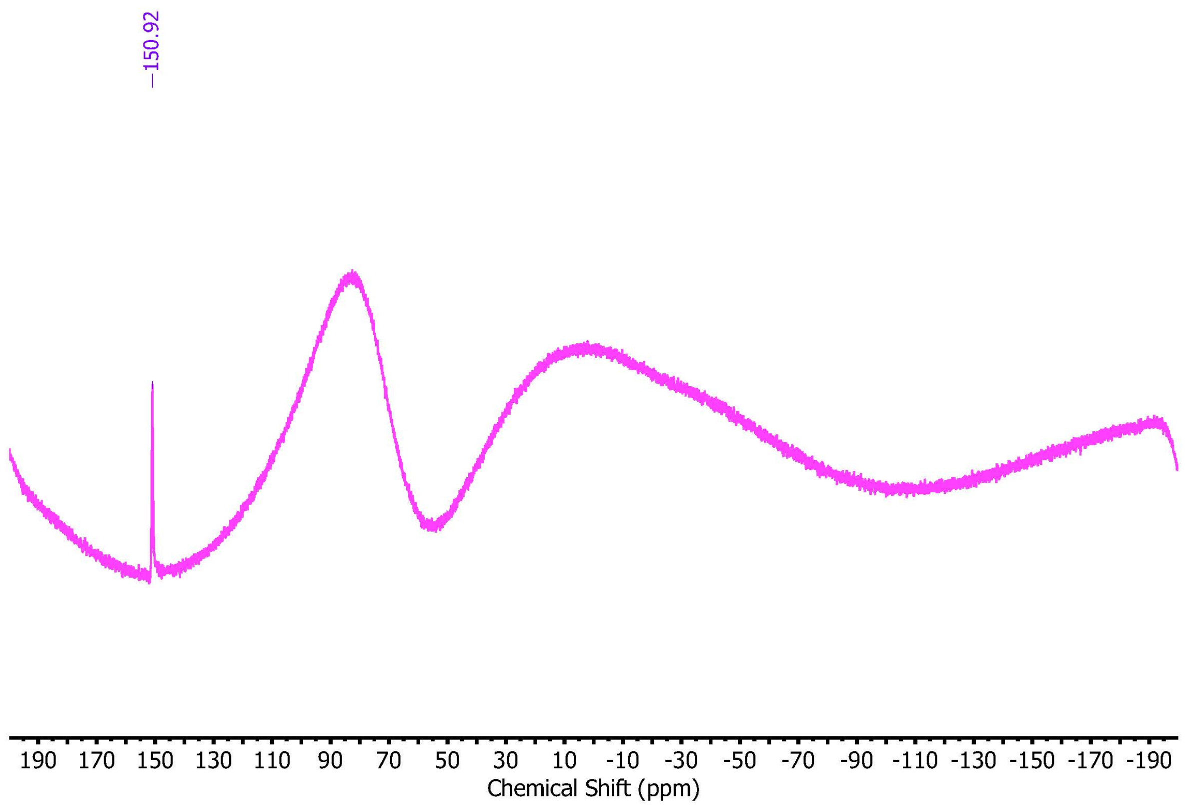

Figure S19 | 104 MHz ^{27}Al NMR of **3-Al** in THF-h_8. Broad background signal appears at about 90 ppm. The baseline from 50 to -200 ppm is uncorrected here to prevent distortion of the **3-Al** peak position.

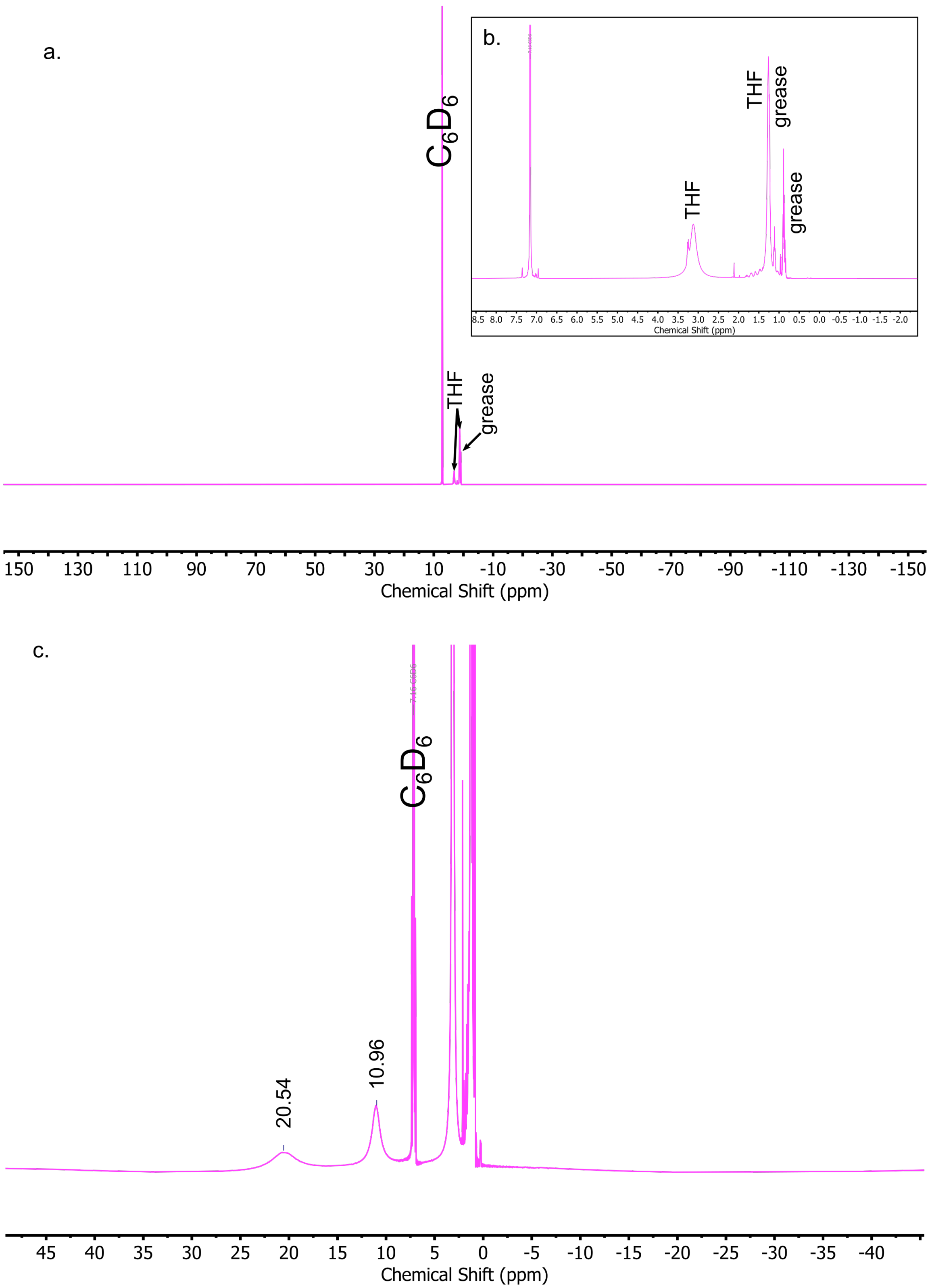

Figure S20 | (a) 400 MHz ^1H NMR of **1** in C_6D_6 with an inset (b) showing the diamagnetic region containing solvent impurities. (c) Paramagnetically shifted peaks of resulting from **1** at ~20.5 and 11 ppm. No peaks associated with free ligand or ligand decomposition could be identified.

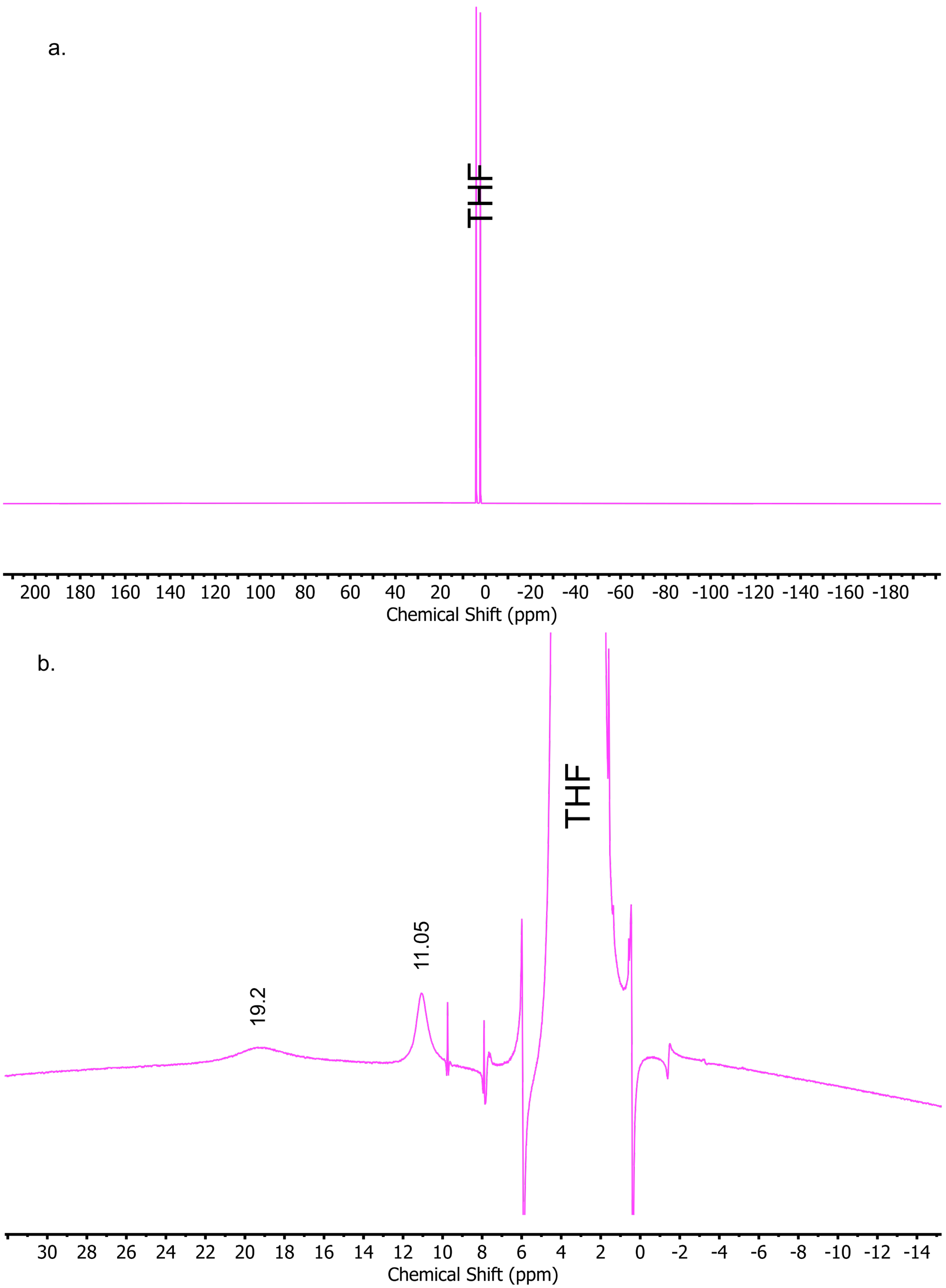

Figure S21 | (a) 400 MHz ^{1}H NMR of **1** in THF-h_8. (b) Paramagnetically shifted peaks at ~19.2 and 11 ppm resulting from the spin-triplet ground state of **1**. Sharp peaks are a resulting of 9.74, 7.87, 5.95, 0.4, and −1.42 ppm are artifacts exacerbated from the measurement in non-deuterated solvent.

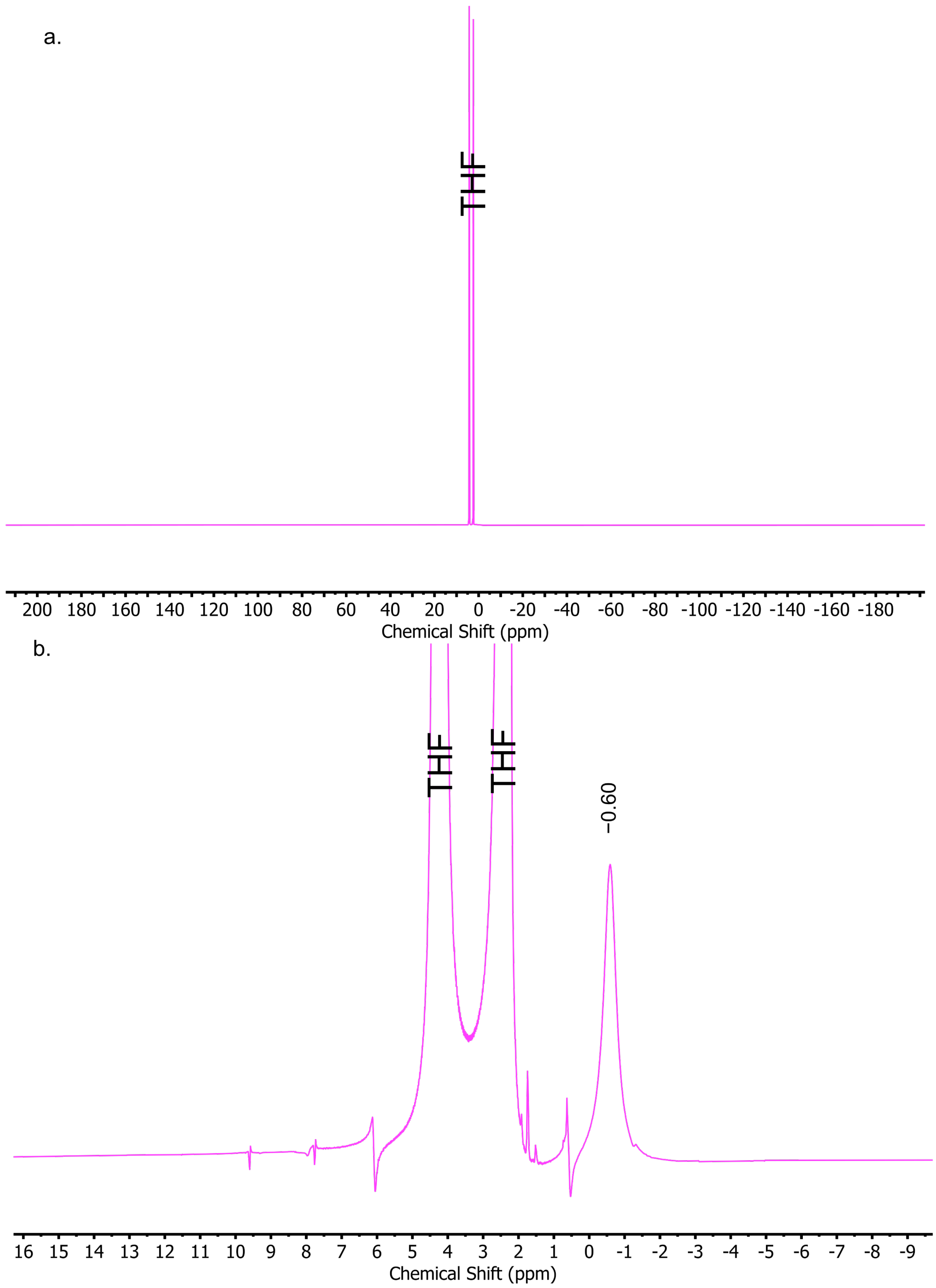

Figure S22 | (a) 400 MHz ^{1}H NMR of **3** in THF-h_8. (b) Paramagnetically shifted peak at −0.60 ppm resulting from the spin-triplet ground state of **3**. Sharp peaks are a resulting of 9.6, 7.8, 6.1, and 0.6 ppm are artifacts exacerbated from the measurement in non-deuterated solvent. No peaks associated with free ligand or ligand decomposition could be identified.

References

(1) Manzer, E. Tetrahydrofuran Complexes of Selected Early Transition Metals. *Inorg. Synth* **1982**, *21*, 135.

(2) Arnold, J.; Wilkinson, G.; Hussain, B.; Hursthouse, M. B. Synthesis and X-Ray Crystal Structure of Tetra (2- Methylphenyl)Molybdenum(Iv), Mo(2-MeC6H4)4. Redox Chemistry of M (2-MeC6H4)4 Compounds of Molybdenum Rhenium, Ruthenium, and Osmium. *J. Chem. Soc. Dalt. Trans.* **1989**, 2149–2153.

(3) Stoffelbach, F.; Saurenz, D.; Poli, R. Improved Preparations of Molybdenum Coordination Compounds from Tetrachlorobis(Diethyl Ether)Molybdenum(IV). *Eur. J. Inorg. Chem.* **2001**, *2001*, 2699–2703.

(4) Fulmer, G. R.; Miller, A. J. M.; Sherden, N. H.; Gottlieb, H. E.; Nudelman, A.; Stoltz, B. M.; Bercaw, J. E.; Goldberg, K. I. NMR Chemical Shifts of Trace Impurities: Common Laboratory Solvents, Organics, and Gases in Deuterated Solvents Relevant to the Organometallic Chemist. *Organometallics* **2010**, *29*, 2176–2179.

(5) Shrestha, B.; Thapa, S.; Gurung, S. K.; Pike, R. A. S.; Giri, R. General Copper-Catalyzed Coupling of Alkyl-, Aryl-, and Alkynylaluminum Reagents with Organohalides. *J. Org. Chem.* **2016**, *81*, 787–802.

(6) Schneider-Koglin, C.; Mathiasch, B.; Dräger, M. Über Tetraaryl-Methan-Analoga in Der Gruppe 14. III. Ar_4Sn/Pb (Ar Ph, p-, m-, o-Tol, 2,4-Xyl Und 2,5-Xyl): Gegenüberstellung von Bindungslängen Und Winkeln, von NMR Chemischen Verschiebungen Und Kopplungskonstanten Und von Schwingungsdaten. *J. Organomet. Chem.* **1994**, *469*, 25.

(7) MATLAB. *MATLAB (R2012b)*; The MathWorks Inc.: Natick, Massachusetts, 2012.

(8) Stoll, S.; Schweiger, A. EasySpin, a Comprehensive Software Package for Spectral Simulation and Analysis in EPR. *J. Magn. Reson.* **2006**, *178*, 42.

(9) OriginLab. *Origin*; Northampton, MA, 2015.

(10) Rigaku. *CrysAlisPro Software System, Version 1.171. 38.41 l, Rigaku Coorporation*; Rigaku Oxford Diffraction, UK, 2015.

(11) Dolomanov, O. V; Bourhis, L. J.; Gildea, R. J.; Howard, J. A. K.; Puschmann, H. OLEX2: A Complete Structure Solution, Refinement and Analysis Program. *J. Appl. Cryst.* **2009**, *42*, 339.

(12) Sheldrick, G. M. SHELXT-Integrated Space-Group and Crystal-Structure Determination. *Acta Cryst. A* **2015**, *71*, 3.

(13) Sheldrick, G. M. Crystal Structure Refinement with SHELXL. *Acta Cryst. C* **2015**, *71*, 3–8.

(14) Kresse, G.; Hafner, J. Ab Initio Molecular Dynamics for Liquid Metals. *Phys. Rev. B* **1993**, *47*, 558–561.

(15) Kresse, G.; Hafner, J. Ab Initio Molecular-Dynamics Simulation of the Liquid-Metal--Amorphous-Semiconductor Transition in Germanium. *Phys. Rev. B* **1994**, *49*, 14251–14269.

(16) Kresse, G.; Furthmüller, J. Efficient Iterative Schemes for Ab Initio Total-Energy Calculations Using a Plane-Wave Basis Set. *Phys. Rev. B* **1996**, *54*, 11169–11186.

(17) Kresse, G.; Furthmüller, J. Efficiency of Ab-Initio Total Energy Calculations for Metals and Semiconductors Using a Plane-Wave Basis Set. *Comput. Mater. Sci.* **1996**, *6*, 15–50.

(18) Kresse, G.; Joubert, D. From Ultrasoft Pseudopotentials to the Projector Augmented-

Wave Method. *Phys. Rev. B* **1999**, *59*, 1758–1775.
(19) Blöchl, P. E. Projector Augmented-Wave Method. *Phys. Rev. B* **1994**, *50*, 17953–17979.
(20) Perdew, J. P.; Burke, K.; Ernzerhof, M. Generalized Gradient Approximation Made Simple. *Phys. Rev. Lett.* **1996**, *77*, 3865–3868.
(21) Perdew, J. P.; Burke, K.; Ernzerhof, M. Generalized Gradient Approximation Made Simple [Phys. Rev. Lett. 77, 3865 (1996)]. *Phys. Rev. Lett.* **1997**, *78*, 1396.
(22) Gavnholt, J.; Olsen, T.; Engelund, M.; Schiøtz, J. Δ Self-Consistent Field Method to Obtain Potential Energy Surfaces of Excited Molecules on Surfaces. *Phys. Rev. B* **2008**, *78*, 75441.
(23) Hellman, A.; Razaznejad, B.; Lundqvist, B. I. Potential-Energy Surfaces for Excited States in Extended Systems. *J. Chem. Phys.* **2004**, *120*, 4593–4602.
(24) Laorenza, D. W.; Kairalapova, A.; Bayliss, S. L.; Goldzak, T.; Greene, S. M.; Weiss, L. R.; Deb, P.; Mintun, P. J.; Collins, K. A.; Awschalom, D. D.; Berkelbach, T. C.; Freedman, D. E. Tunable Cr^{4+} Molecular Color Centers. *J. Am. Chem. Soc.* **2021**, *143*, 21350–21363.
(25) Figgis N., B.; Hitchman A., M. Tanabe-Sugano Diagrams. In *Ligand Field Theory and Its Applications*; Wiley-VCH: New York, 2000; pp 131–141.
(26) Dobrovitski, V. V.; Fuchs, G. D.; Falk, A. L.; Santori, C.; Awschalom, D. D. Quantum Control over Single Spins in Diamond. *Annu. Rev. Condens. Matter Phys.* **2013**, *4*, 23–50.
(27) Doherty, M. W.; Manson, N. B.; Delaney, P.; Jelezko, F.; Wrachtrup, J.; Hollenberg, L. C. L. The Nitrogen-Vacancy Colour Centre in Diamond. *Phys. Rep.* **2013**, *528*, 1–45.
(28) Salikhov, K. M.; Dzuba, S. A.; Raitsimring, A. M. The Theory of Electron Spin-Echo Signal Decay Resulting from Dipole-Dipole Interactions between Paramagnetic Centers in Solids. *J. Magn. Reson.* **1981**, *42*, 255–276.
(29) Wolfowicz, G.; Tyryshkin, A. M.; George, R. E.; Riemann, H.; Abrosimov, N. V.; Becker, P.; Pohl, H. J.; Thewalt, M. L. W.; Lyon, S. A.; Morton, J. J. L. Atomic Clock Transitions in Silicon-Based Spin Qubits. *Nat. Nanotechnol.* **2013**, *8*, 561–564.
(30) Kugelgen, S. Von; Krzyaniak, M. D.; Gu, M.; Puggioni, D.; Rondinelli, J. M.; Wasielewski, M. R.; Freedman, D. E. Spectral Addressability in a Modular Two Qubit System. *J. Am. Chem. Soc.* **2021**, *143*, 8069–8077.
(31) Yang, L.; Powell, D. R.; Houser, R. P. Structural Variation in Copper(i) Complexes with Pyridylmethylamide Ligands: Structural Analysis with a New Four-Coordinate Geometry Index, T4. *J. Chem. Soc. Dalt. Trans.* **2007**, No. 9, 955–964.
(32) Okuniewski, A.; Rosiak, D.; Chojnacki, J.; Becker, B. Coordination Polymers and Molecular Structures among Complexes of Mercury(II) Halides with Selected 1-Benzoylthioureas. *Polyhedron* **2015**, *90*, 47–57.
